# Mitral Annular Disjunction: A Roadmap for the Surgeon

**DOI:** 10.1093/ejcts/ezaf461

**Published:** 2025-12-17

**Authors:** Nikolaos Bonaros, Can Gollmann-Tepeköylü, Meindert Palmen, Nina Ajmone, Victoria Delgado, Madalina Garbi, Agnes Mayr, Leo Pölzl, Mateo Marin-Cuartas, Guido Ascione, Nicolo Azzola Guicciardi, Felix Troger, Daniel Pereda, Robert Klautz, Michele De Bonis, Michael Andrew Borger, Patrick Perier

**Affiliations:** Department of Cardiac Surgery, University Hospital Innsbruck, Medical University of Innsbruck, A-6020, Innsbruck, Austria; Department of Cardiac Surgery, University Hospital Innsbruck, Medical University of Innsbruck, A-6020, Innsbruck, Austria; Department of Cardiothoracic Surgery, Leiden University Medical Center, 2300, Leiden, The Netherlands; Department of Cardiology, Leiden University Medical Center, 2300, Leiden, The Netherlands; Heart Institute, University Hospital Germans Trias i Pujol, and Center of Comparative Medicine and Bioimaging, 08916, Badalona, Spain; Department of Cardiology, Royal Papworth Hospital Royal Papworth Hospital NHS Foundation Trust, Cambridge Biomedical Campus, CB2 0AY, Cambridge, United Kingdom; Department of Radiology, University Hospital Innsbruck, Medical University of Innsbruck, A-6020, Innsbruck, Austria; Department of Cardiac Surgery, University Hospital Innsbruck, Medical University of Innsbruck, A-6020, Innsbruck, Austria; University Department of Cardiac Surgery, Leipzig Heart Center, D-04289, Leipzig, Germany; Department of Cardiac Surgery, IRCCS San Raffaele Scientific Institute, 20132, Milan, Italy; Department of Cardiac Surgery, IRCCS San Raffaele Scientific Institute, 20132, Milan, Italy; Department of Radiology, University Hospital Innsbruck, Medical University of Innsbruck, A-6020, Innsbruck, Austria; Department of Cardiovascular Surgery, Hospital Clínic, 08036, Barcelona, Spain; Department of Cardiothoracic Surgery, Leiden University Medical Center, 2300, Leiden, The Netherlands; Department of Cardiac Surgery, IRCCS San Raffaele Scientific Institute, 20132, Milan, Italy; University Department of Cardiac Surgery, Leipzig Heart Center, D-04289, Leipzig, Germany; Department of Cardiovascular Surgery, Campus Bad Neustadt, D-97616, Bad Neustadt/Saale, Germany

**Keywords:** mitral regurgitation, mitral repair, valve surgery, ventricular arrhythmias

## Abstract

**Objectives:**

Mitral annular disjunction (MAD) is a structural abnormality of the mitral annulus fibrosus, associated with myxomatous leaflet degeneration, mitral valve prolapse (MVP), and ventricular arrhythmias. The combination of annular dilatation and abnormal annular motion increases mechanical stress on the mitral leaflets, triggering the degenerative process.

**Methods:**

This review summarizes the major pathophysiologic, diagnostic and therapeutic measures for the treatment of patients with MAD and an indication for mitral surgery.

**Results:**

The diagnosis is primarily based on non-invasive imaging techniques. Echocardiography is the first choice due to its ability to assess real-time mitral valve function. Cardiac computed tomography and magnetic resonance imaging provide more detailed information on the extent of MAD and the presence of calcifications. Indications for surgical mitral valve treatment are based on current recommendations. In cases with MAD and moderate mitral regurgitation, early intervention may be advocated in the presence of arrhythmogenic MVP. Long-term outcomes after treatment are assessed through multimodal imaging and electrocardiogram monitoring. A ring annuloplasty is an important cornerstone of treatment. Stabilization of the mitral annulus abolishes functional prolapse and increases the antiarrhythmic effect of mitral surgery. However, postoperative arrhythmic burden may persist in some cases, requiring continuous monitoring and sometimes an additional device therapy.

**Conclusions:**

MAD represents a complex anatomical and functional entity associated with diagnostic challenges and rhythm abnormalities. Although the current indications for surgical treatment follow the recommendations for treatment of primary mitral regurgitation, early treatment may be important especially in patients with arrhythmias.

## Graphical abstract

**Figure:**
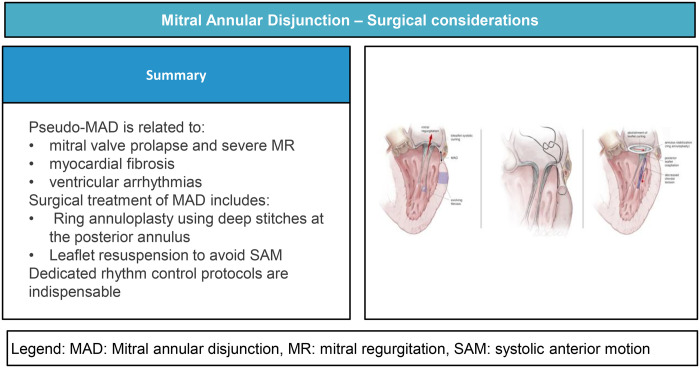

## Introduction

**Figure 1.**
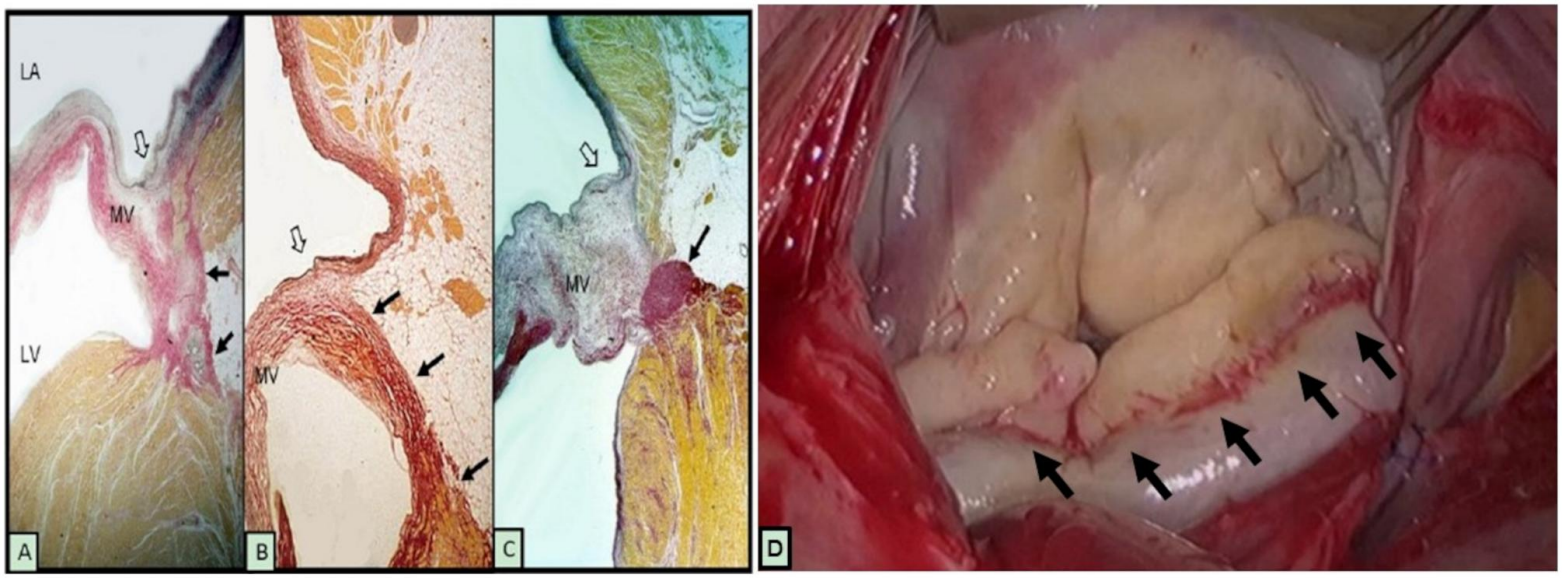
Typical characteristics of mitral annular disjunction. Posterior leaflet attachment to mitral annulus (open arrows). attachment of the posterior leaflet to fibrous tissue of the atrioventricular junction (black arrows) in a normal valve (A and B). Myxomatous posterior mitral leaflet hinged to the atrial wall and a cord-like annulus (Arrow). Reproduced with permission from Wunderlich et al.^2^ Typical characteristics of mitral annular disjunction with excessive valve tissue, mucoid degeneration affecting the posterior leaflet (C). Note the signs of fibrosis (black arrows) detected at the area of maximal annular mobility (D). LA, left atrium; LV, left ventricle; MV, mitral valve.

The mitral annulus can be defined as the hinge line between the mitral leaflets and the atrioventricular junction. While the anterior annulus is anatomically defined by the fibrous tissue between the left and right fibrous trigones, the attachment of the posterior mitral leaflet to the hinge varies significantly.^1^ The anterior annulus is commonly characterized by thick and well organized fibrous structures, producing a cord-like segment of ring. However, in the posterior part, the hinge of the leaflet maybe atrially displaced and is not always connected to the atrioventricular junction. The posterior annulus can develop as a cord-like fibrous tissue, as a thin curtain-like structure, or can be covered by adipose tissue (**Figure 1A and B**).^2^ The laxity of the mitral annulus and the suspension of the leaflets to the atrioventricular areas are likely key factors for progressive annular dilation and the characteristic pathological annular motion.

Mitral annulus disjunction (MAD) is a structural abnormality of the annulus fibrosus defined as a distinct separation between the insertion of the posterior mitral valve (MV) leaflet at the level of the left atrium and the ventricular myocardium.^3^ In addition, the annulus expands and flattens during systole and the left ventricular wall moves outwards in relation to the annulus. This motion increases the mechanical stress on the leaflets and the tendinous chords and is presumed to trigger the degenerative process of the MV. In some cases, an extensive MAD will result in generalized excess of MV tissue, mucoid degeneration and bi-leaflet prolapse, all of which are typical characteristics of Barlow’s disease (**Figure 1C**). On the contrary a restricted MAD will be responsible for a focal posterior leaflet prolapse with limited area of mucoid degeneration. It is important to understand that some degree of MAD exists in normal MV.

True MAD refers to an anatomical disjunction between the insertion site of the mitral leaflet at the atrial endocardium and the ventricular myocardium, present in both diastole and systole, and not associated with severe MR. It may involve the anterior and the posterior annulus and has been identified in up to 96% of the normal population.^4^ Importantly, extension of MAD into the paracommissural areas is considered as a normal variant without clinical implications. Pseudo-MAD refers to an apparent separation of the atrioventricular junction that occurs only during systole, caused by a billowing leaflet and the systolic curling of the ventricular myocardium. The latter results in a relative prolapse of the posterior or both mitral leaflets and is associated with MVP.^5^

**Figure 2.**
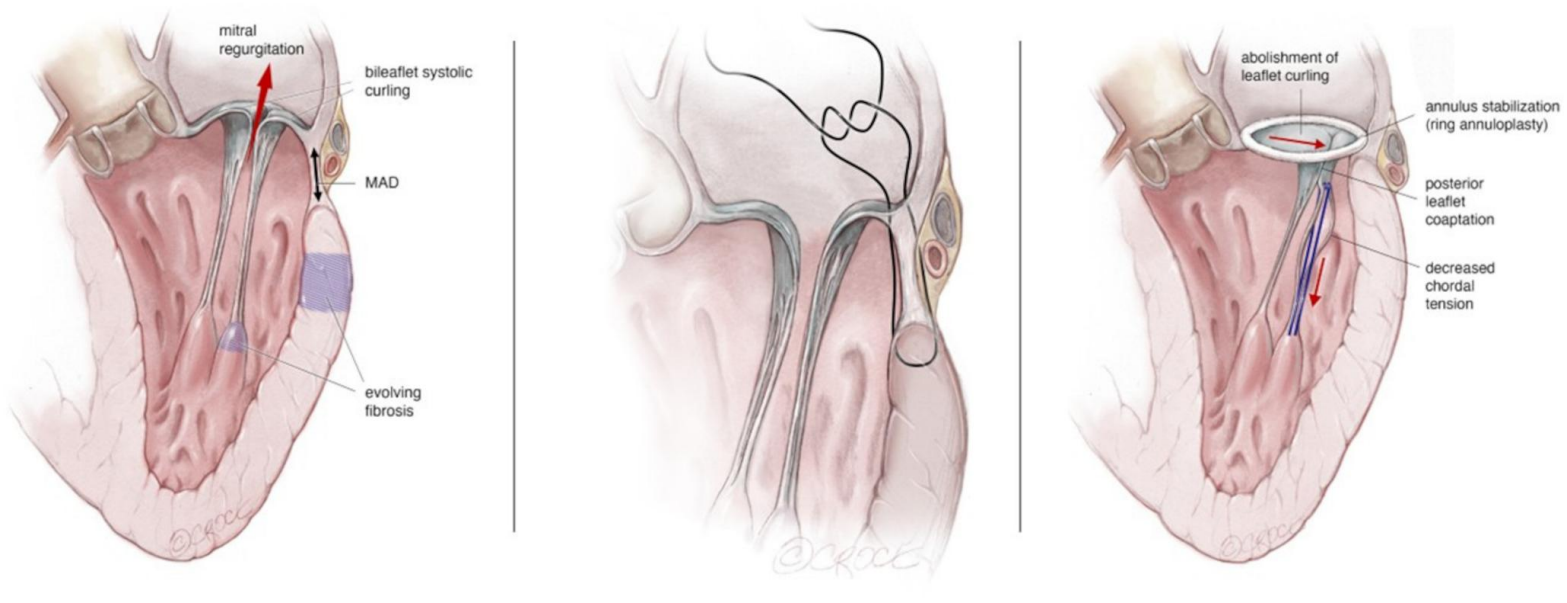
Surgical treatment of MAD. Evolving myocardial fibrosis due to mechanical stress (left). Use of deep annular stitches to stabilize the annulus (middle). Use of artificial chordae to reduce the mechanical stress on the leaflet.

Over time, mechanical stress on the leaflet and the subvalvular apparatus triggers a proliferative cascade which is characterized by compensatory growth and thickening of MV leaflets and the chordae tendinae. In case of extensive MAD, when annular dilation progresses beyond the compensatory capacity of leaflet growth, coaptation is lost, leading to central MV regurgitation.^6^ At this stage, there is still no true anatomical prolapse but rather a “functional prolapse” which results from a combination of bi-leaflet curling and systolic annulus dilatation.^7^ With time, excess tension and mechanical stress on the chordae tendinae, especially of the posterior leaflet, may cause their elongation or rupture, producing a subsequent anatomical prolapse or flail.^8^ The mechanical stress imposed on the subvalvular apparatus and the mitral annulus may lead to focal fibrosis of the papillary muscles and/or the ventricular myocardium (**Figure 2**). This is considered a potential contributor to ventricular arrhythmias connected to the arrhythmogenic MV syndrome.^9^ In clinical practice, MAD is associated with myxomatous leaflet degeneration, MV prolapse (MVP), and ventricular arrhythmias.

## Diagnostic criteria of MAD

The diagnosis of MAD is mainly based on non-invasive imaging (**Table 1**), which, unlike the mere surgical inspection, can provide valuable functional information. Echocardiography remains the first-line imaging modality due to its wide accessibility and ability to assess real-time MV function. Cardiac computed tomography (CT) and magnetic resonance imaging (CMR) play complementary roles, offering enhanced spatial resolution and detailed anatomical information.

**Table 1.** Comparison of Imaging Modalities for the Diagnosis of MAD

Modality	Criteria	Strengths	Limitations
**TTE/TOE**	MAD best visualized in parasternal or mid-oesophageal views and both in systole and diastole to exclude pseudo-MAD; Minimum systolic separation minimum >2 mm.	Widely available, real-time imaging/functional evaluation	Operator dependence, limited acoustic windows
**CT**	MAD defined as systolic separation >3 mm on gated images, thin-slice reconstruction of LV long-axis view	High spatial resolution, detailed anatomic characterization	Ionizing radiation, contrast exposure
**CMR**	MAD defined as systolic separation >2 mm on long axis views; dynamic cine evaluation to exclude pseudo-MAD	Tissue characterization, no radiation, superior dynamic assessment	Higher cost, longer acquisition time, limited availability and expertise

### Echocardiography

**Figure 3.**
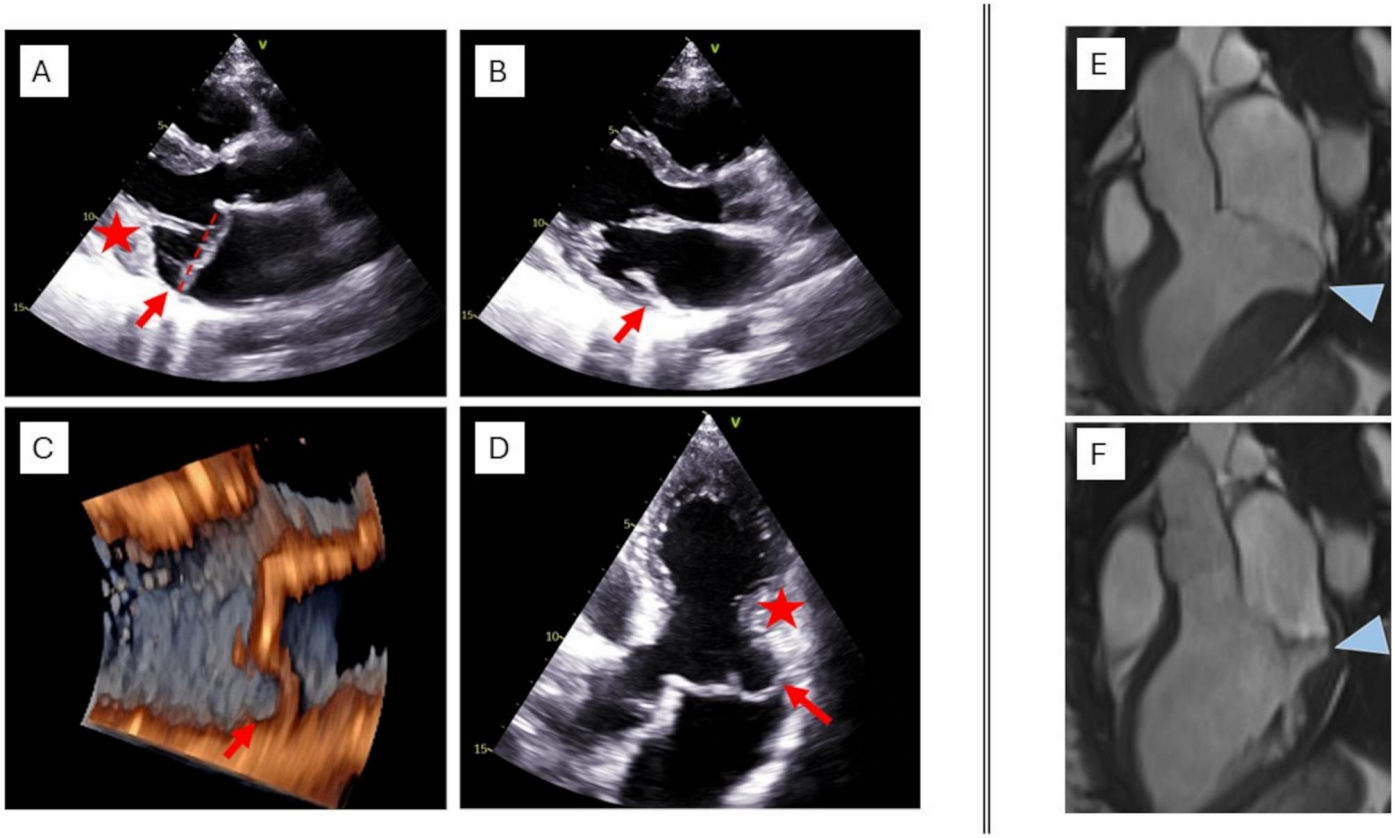
Mitral annular disjunction (red arrows) identified in systole (A) but not clearly visualized in diastole (B). The star indicates a posterolateral, basal myocardial hypertrophy. The dotted line indicates the MV annulus level. Another example of MAD (red arrows) visualized by 3D TTE (C) and confirmed in an apical 3-chamber view (D). Assessment of MAD by CMR (E, F). Cine three-chamber views of a patient with MAD of the P2-segment (arrowheads) measuring 11 mm in systole, demonstrating how MAD can be better assessed at end-systole (E) as compared to end-diastole (F).

MAD can be identified by transthoracic echocardiography (TTE) as an atrial insertion of the posterior MV leaflet. It is mainly visualized in systole from a 2-dimensional parasternal long-axis view with the highest frame rate possible (**Figure 3A and 3B**): its length is measured as the maximal distance between the left atrial wall-mitral annular plane and the bulging LV myocardium.^9^ Measurements > 2 mm are considered diagnostic, although thresholds of 3–5 mm may improve specificity. Mid-oesophageal long-axis view from trans-oesophageal echocardiography are used to provide the same measurement in borderline cases or suboptimal TTE quality (**Figure 3**). However, MAD is rarely focal and its circumferential *extension* along the posterior MV annulus should be also explored using 2-, 3-, and 4-chamber apical views. With this purpose, 3D echocardiography can also be performed to identify the presence of multisegmental MAD^10^ (**Figure 3C**).

Although MAD is measured in systole, frame-by-frame dynamic examination of the images including diastole, should be performed, as a true MAD is present throughout the entire cardiac cycle. On the contrary, pseudo-MAD is identified only during systole, and since there is no true displacement of leaflet insertion, it can be observed only in the presence of MV prolapse.^5^ It is important to note that in presence of MAD, MV annulus level is in principle redefined at the level of leaflet insertion^9^ (**Figure 3D**).

Importantly, other MV annulus abnormalities accompany MAD and should be systematically described. Of particular importance is the identification of “curling”, as a systolic outward and apical motion of the LV basal myocardium, which results in a mid-late systolic abnormal annular expansion worsening mitral regurgitation (MR).^10^ In some cases, also focal annular calcifications are observed. Notably in case of repeated examinations, the evaluation of MAD and related annulus abnormalities should be reported over time and might be useful to evaluate progression and relation to clinical outcome.^6^

### Cardiac computed tomography

Cardiac CT allows for structural and anatomic characterization in MAD. However, the requirement for ionizing radiation and contrast media currently limits its use. It provides exceptional spatial resolution, facilitating precise delineation of the MV annular anatomy and adjacent structures, as well as the presence of calcificiations; The technique allows for quantification and localization of calcified tissue, and differentiation between fibrosis and real calcifications if echocardiography is inconclusive. Thin-slice reconstructions (≤0.5 mm) and multiplanar reformations are critical for accurate measurement. Retrospective ECG-gated CT acquisitions capture the dynamic motion of the mitral annulus, although the assessment of MAD in diastole (and therefore the distinction between “true” and “pseudo-MAD”) remains challenging.

### Cardiac magnetic resonance imaging

CMR is well-suited to assess the presence and extension of MAD thanks to the good spatial resolution and the optimal contrast between myocardium and blood pool. This technique would also provide important information on chamber volume quantification. It also enables comprehensive myocardial tissue characterization, including detection of late gadolinium enhancement (LGE), and increased values of myocardial T1 relaxation times and extracellular volume.^11^ However, due to the limited availability and to the lack of expertise, this imaging modality is typically underused.

By CMR, MAD is ideally assessed using balanced steady-state free precession cine sequences acquired by using breath-hold and retrospective ECG-triggered sequences, a temporal resolution <45 ms between phases and with flip angles of 50°–80°.^12^^,^^13^ Longitudinal MAD length is best measured at end-systole in long-axis views (**Figure 2E**), while to determine the circumferential MAD extent, respective basal short-axis views shall be used.^14^ Up to now, there is no uniform CMR threshold definition of MAD, with some authors reporting any extent of disjunction, disjunction >1 mm, >2 mm^15^ or even >5 mm^16^ (**Figure 2F**).

## Indications and timing of surgery

Indications for MV treatment are based on the current recommendations for the treatment of MV disease.^17^ According to them, the presence of MAD without severe MR even in the context of ventricular arrhythmias is not considered to be an important indications modifier. Nevertheless, based on the natural history of the disease, the majority of the patients with moderate MR and severe MAD are exposed to increased tension forces at the level of the primary chordae and the mitral annulus leading to elongation and rupture. In addition, long-lasting moderate MR may worsen during exercise leading to subsequent dilatation of the mitral annulus. Therefore, in most of the patients with severe MAD, moderate MR will deteriorate to severe earlier than in other types of chronic MR.^6^ However, the duration of the transition phase may vary significantly. To illustrate the different phases of the disease and the indication for surgical treatment we present 3 different scenarios in patients with MAD and MR.

### Scenario #1: Patient with MAD and severe MR

**Figure 4.**
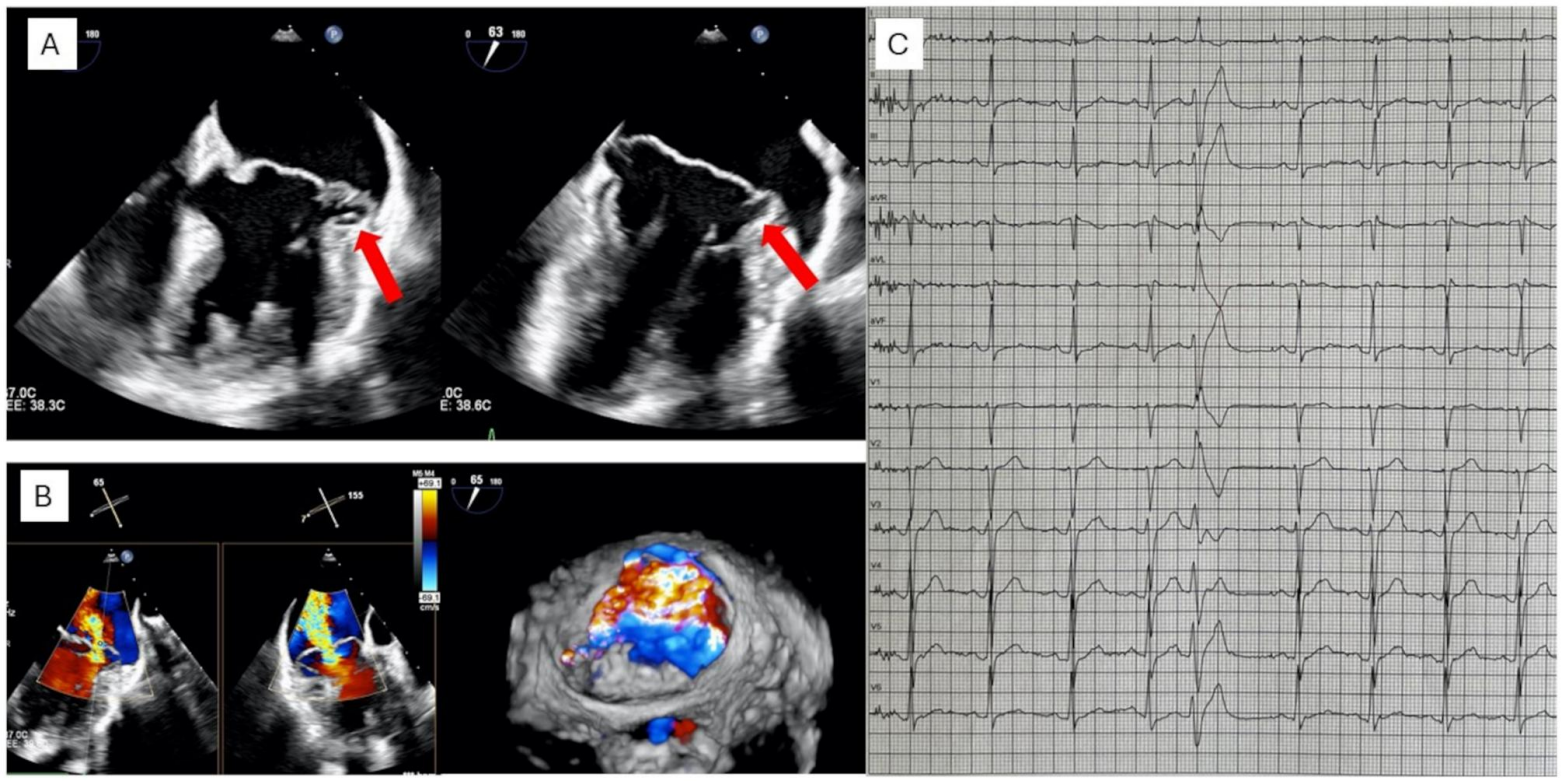
A Patient with MAD and severe MR. Pre-operative TOE showing a severely myxomatous mitral valve (A), with bi-leaflet prolapse and pseudo-MAD (arrow) as well as jets location (B). ECG showing normal sinus rhythm with a premature ventricular contraction originating from posterior papillary muscle (C).

A 34-year-old woman was referred for severe MR with MAD in the context of bi-leaflet prolapse. The first diagnosis of MVP occurred 3 years prior, during a pre-pregnancy cardiological screening, and had gradually progressed to severe MR accompanied by New York Heart Association class II dyspnoic symptoms at the time of referral. Transoesophageal echocardiography showed a Barlow’s disease, with bi-leaflet prolapse and pseudo-MAD (13 mm) (**Figure 4**). MR was severe and characterized by multiple meso-telosystolic jets, with the largest regurgitant jet located centrally (A2-P2) and at the anterolateral commissure (A1-P1). A 24-h Holter ECG revealed few premature ventricular beats (posterior papillary muscle morphology) and excluded atrial fibrillation or other ventricular arrhythmias. In this case, the indication for surgery is based on the treatment of symptomatic severe primary MR due to bi-leaflet prolapse in a Barlow valve with MAD to improve symptoms and prognosis. At this stage, it remains unknown whether the treatment of mitral disease affects premature ventricular extrasystoles.

### Scenario #2: Patient with MAD, mitral annular calcifications, severe MR, arrhythmogenic MVP and out of hospital cardiac arrest

A 77-year-old male patient was admitted to the emergency department after out of hospital cardiac arrest. After initial resuscitation and defibrillation of a ventricular fibrillation with an automatic external defibrillation, spontaneous circulation was restored. TTE revealed pseudo-MAD (17 mm) with severe primary MR on the grounds of primary chordae rupture and posterior leaflet flail due to Barlow’s disease. CMR revealed focal late gadolinium enhancement in the posterior papillary muscle verifying focal fibrotic spots, while CT demonstrated areas of posterior mitral annulus calcifications (MAC). Myocardial fibrosis has been recognized as a trigger of ventricular arrhythmias. The patient was scheduled for urgent MV repair with annular debridement. In this case, the indication of urgent treatment is based on the treatment of the mitral pathology along with the possibility to reduce potential triggering of life-threatening arrhythmias by mechanical stress related to MAD. Apart from that, implantable cardiac defibrillator (ICD) implantation was indicated as secondary prophylaxis to address the residual risk of ventricular arrhythmias due to detected myocardial fibrosis (**Figure S1**).

### Scenario #3: Patient with MAD and moderate to severe MR

**Figure 5.**
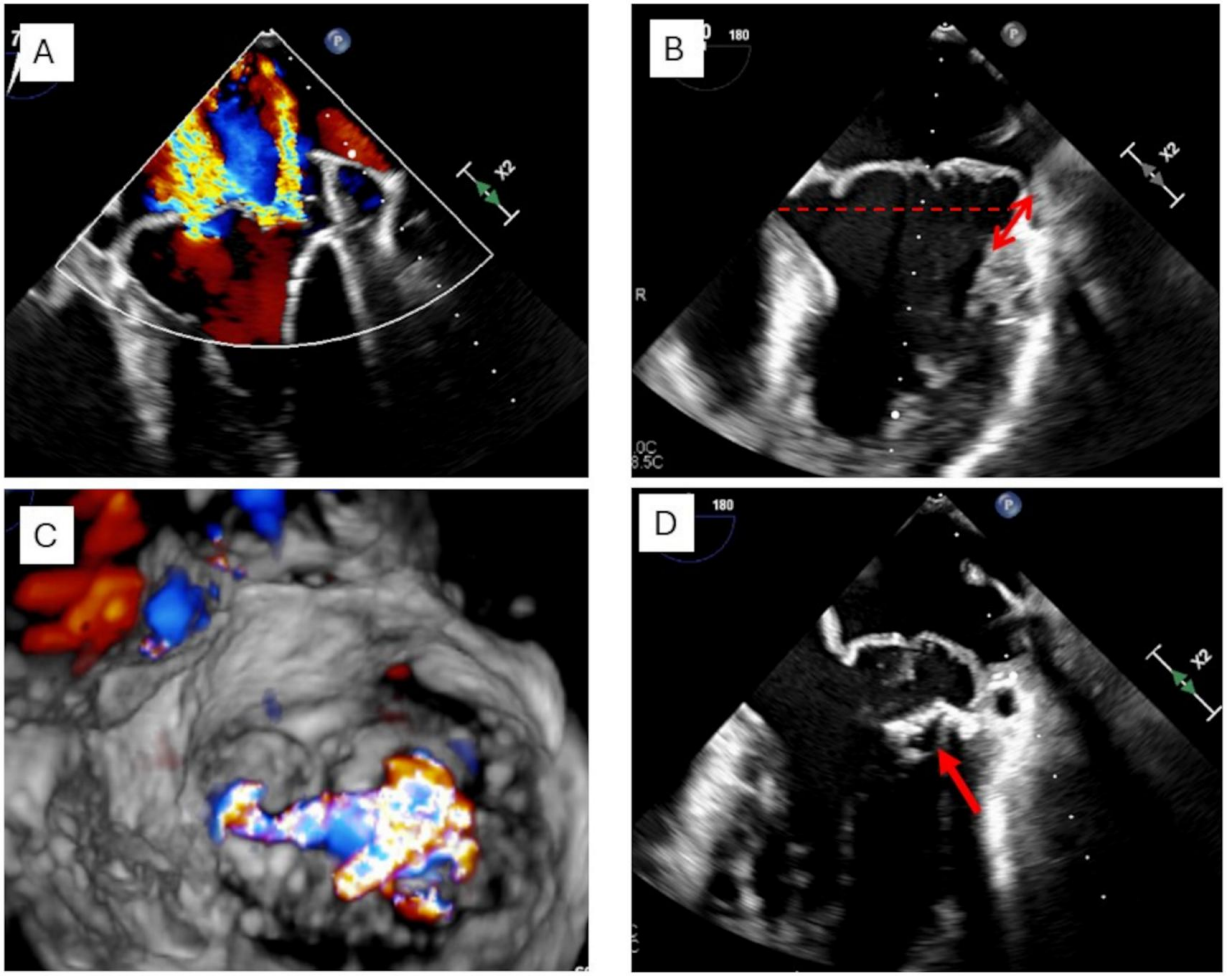
Patient with MAD and Symptomatic Moderate to Severe MR and History of Palpitations. Presence of 2 central jets related to a myxomatous degeneration (A), bi-leaflet prolapse (dotted line) and mitral annular disjunction (arrow) (B) with a wide loss of coaptation at the end-systole (C). Severe annular and subannular calcification (arrow) at the lateral commissure and the posterior annulus corresponding to P1 and lateral P2 segment (D).

A 43-year-old male patient with moderate MR and Barlow’s disease has been followed in an outpatient clinic on a yearly basis for the last 4 years. During the last follow-up, the patient mentioned increased symptoms of exhaustion and palpitations. The echocardiographic examination revealed a progression from moderate to severe MR on the grounds of a functional bi-leaflet prolapse. There were signs of left ventricular dilatation (LVESD = 45 mm) and systolic left ventricular dysfunction (EF = 43%). Two ancestors in patient’s family had a history of sudden cardiac death and ventricular arrhythmias without connection to a known mitral disease (aunt died at the age of 55, mother survived an episode of ventricular fibrillation at the age of 50). MV surgery was indicated for the treatment of progressive (from moderate to severe) MR to improve symptoms and prognosis due to left ventricular dysfunction and dilatation. Genetic assessment excluded channelopathies or other known hereditary forms of syndromes associated with ventricular arrhythmias (**Figure 5**).

The aforementioned scenarios underscore the importance of identifying MR related to MAD at an early stage and before the development of anatomical and functional complications. These complications may range from life-threatening arrhythmias to severe annular calcifications, both of which significantly affect the success of mitral repair and patient’s prognosis. To date, indications for intervention are limited to patients with severe MR. However, close echocardiographical and clinical monitoring are essential for early detection of disease progression, which is crucial for identifying potential candidates for early surgery, even before the onset of symptoms.

## Repair techniques in MAD: rationale and patient selection

Patients with severe primary MR and MAD may be referred to surgery at different stages of the disease. Understanding the pathophysiological mechanisms involved in the development of MR in patients with MAD is of paramount importance for achieving a successful and durable surgical result. Pre-operative echocardiography and intraoperative surgical valve analysis are crucial in guiding surgical repair. Specific lesions not revealed during echocardiography (ie deep indentations between scallops, leaflet perforations, leaflet prolapse masked by prolapse of opposite leaflet segments and focal infiltration/calcification of the leaflet and the annulus,) should be systematically assessed.

MAD is commonly presented as annular fibrosis (**Figure 1D**) and in combination with Barlow’s disease. Determining the extent and location of annular fibrosis or calcification, myxomatous degeneration and the presence of jet lesions will guide the surgeon in addressing the affected part of the valve. Subsegmental analysis of leaflet pliability and mobility in relation to a non-prolapsing reference point, along with assessment of excess leaflet tissue in height and width is crucial for creating an adequate surface of coaptation and preventing of systolic anterior motion (SAM). Functional prolapse is a condition closely associated to MAD and can be recognized by the almost complete abolishment of MR at water test prior to the beginning of any repair measures (**Video 1**). Finally, the anatomy of the subvalvular apparatus is highly variable and may present with distinct lesions (chordae elongation or rupture) that need to be addressed during MV repair.

## Annuloplasty-only in patients with functional prolapse

MAD is associated with a pathological leaflet motion caused by excessive movement of the posterior annulus. Therefore, remodelling and stabilizing the mitral annulus during MV repair is therefore essential for the treatment of MAD, regardless of the leaflet repair technique used. In cases presented with a central regurgitation jet, indicating the absence of additional leaflet pathology, annuloplasty alone can correct the functional prolapse by eliminating abnormal and excessive annular motion and addressing potential annular dilation (**Video 2**). This is achieved by reducing the septo-lateral annular diameter and creating a broad surface of leaflet-to-leaflet coaptation, as in most cases of chronic MR. The use of deep annuloplasty stiches particularly along the posterior annulus, aims to engage the base of the left ventricular myocardium and reduce the atrioventricular gap, which is characteristic for MAD (**Figure 2**). This technique stabilizes the mitral annulus in an early systolic frame. At an early disease stage, annuloplasty alone may restore MV competence. Increased recognition and understanding of the concept of functional prolapse have simplified the repair strategy in patients with MAD and Barlow’s phenotype. Consequently, in these patients, anterior leaflet billowing usually does not require direct intervention, as annuloplasty resolves the functional component of leaflet curling.^18^ However, the coaptation line may shift anteriorly after repair, predisposing to SAM.^19^ In such cases, additional posterior leaflet repair techniques may be required to reduce effective height.

## Annuloplasty with posterior leaflet repair in patients with functional prolapse

In patients presented at early stage with functional prolapse and MAD, the risk of SAM should be carefully evaluated. In the subgroup of patients with high risk of SAM, it is crucial to reduce the height of the posterior leaflet, moving the closure line posteriorly, away from the Left Ventricular Outflow Tract (LVOT) and towards the inflow portion of the LV. To accomplish this goal and depending on the amount of excess tissue in width and height, the surgeon can use resectional techniques (i.e. quadrangular resection and leaflet sliding technique, butterfly technique,^20^ resection of the base of the posterior leaflet, foldoplasty^21^) or techniques respecting the posterior leaflet. When choosing to respect, either verticalization of the posterior leaflet, by implanting shortening neochords,^22^ or by using the so-called snail technique^23^ can be used (**Figure 2**). Both resectional and non-resectional techniques are equally efficient in obtaining excellent early long-term outcomes in terms of repair rate and repair durability.^24^^,^^25^

## Annuloplasty with posterior leaflet repair in patients with anatomical posterior leaflet prolapse or flail

In patients with MAD-associated mitral disease at a more advanced stage, true prolapse of posterior leaflet, due to chordal elongation or rupture may develop. Both conditions will result in an eccentric jet on echocardiography, characteristic of an asymmetric Barlow valve with severe MR. In addition to the obligatory annular remodelling,^26^ all above-mentioned surgical techniques can be applied to treat MVP. In recent years, a gradual shift in paradigm of posterior leaflet repair is observed^22^; due to the easy application of neochords in minimally invasive procedures, surgeons tend to use more artificial chordae rather than resection techniques. Many studies have demonstrated that repair rates and durability are comparable for both techniques.^25^^,^^27^^,^^28^

## Annuloplasty with leaflet repair in patients with anatomical anterior leaflet prolapse or flail

The need to address the anterior leaflet in patients with bi-leaflet curling and functional prolapse has been abandoned overtime if no true elongated or ruptured chordae are found in valve analysis.^18^ Obviously, a true prolapse of the anterior leaflet should always be corrected. While in posterior leaflet prolapse, resection is a commonly applied repair technique, it is infrequently performed at the anterior leaflet. Chordal replacement, either using artificial or autologous chords (chordal transfer) are the most common repair strategies.^29^ Chordal replacement to address anterior leaflet prolapse may be more prone to early and mid-term repair failure when compared to correction of the posterior leaflet prolapse.^30^^,^^31^ This may be due to the forces applying on the anterior leaflet and the narrow margin between restriction and residual prolapse.

One of the difficulties of correcting anterior leaflet prolapse using artificial chords is the adjustment of the length of the chords. Most surgeons use the anterior annulus as a reference. Using pre-measured loops referenced on the length of adjacent autologous chords is a well reproducible alternative. When using this technique, it is paramount to secure several loops at the prolapsing part of the anterior leaflet to evenly distribute forces. Finally, the choice of annuloplasty device may be crucial in those patients. The use of a complete ring rather than an open band, allows for anterior annulus stabilization, which is needed to obtain sufficient leaflet coaptation.^32^

## Annuloplasty with edge-to-edge repair

The edge-to-edge technique, has been initially introduced for the treatment of primary MR and represents an important tool for patients with MAD and Barlow phenotype, especially at the presence of complex pathologies (ie, bi-leaflet, anterior leaflet or commissural prolapse). The combination of edge-to-edge repair and ring annuloplasty leads to complete abolishment of MAD, durable treatment of leaflet prolapse and abolishment of SAM. Additionally, it serves as a bailout option in cases of suboptimal immediate result of conventional mitral repair techniques.^33^ It must be emphasized that any form of edge-to-edge repair, whether surgical or transcatheter, performed without the addition of annuloplasty fundamentally fails to address the primary mechanism of mitral disease, which lies in MAD and annular hypermobility. The absence of annuloplasty inevitably leads to persistent mechanical stress on the primary chordae, promoting their progressive elongation and eventual rupture, and thereby predisposing to recurrent or residual MR.

A successful application of the technique hinges on adhering to key principles, such as limiting the suture to the zone of the primary regurgitant jet (central or commissural), employing a horizontal mattress suture followed by a running suture, and respecting leaflet symmetry to avoid distortion (**Video 3**). Of note, the distance of the sutures from the free edge is adjusted based on leaflet redundancy, ensuring an effective and balanced repair.

Despite its broad applicability, contraindications to the edge-to-edge technique include those scenarios with high probability of developing mitral stenosis such as MAD with leaflet or severe annular calcifications. Moreover, it is not recommended in cases of non-localized regurgitant jet and multiple mitral lesions involving non-facing segments of the anterior and posterior leaflets. Finally, it should be avoided whenever annular dilatation cannot be corrected by a concomitant annuloplasty, as the absence of annuloplasty is likely associated with early failure of the repair.^34^ Indeed, both complete rings and posterior bands, in the context of Barlow disease, have shown similar outcomes in terms of effectiveness and durability.^35^^,^^36^

## Selection of annuloplasty ring

Annuloplasty remains one of the mainstays of MV repair. In contrast to conventional cases of primary MR, the principal objective of annuloplasty in patients with MAD is the correction of annular hypermobility, whereas the reduction of annular dilatation and the increase of leaflet coaptation serve as secondary goals. Stabilizing the posterior annulus constitutes a critical component of surgical management of MAD, as it prevents systolic curling of the posterior leaflet and subsequent residual prolapse. Therefore, the use of deep annuloplasty stitches engaging the ventricular myocardium is of paramount importance for the effective elimination of MAD. While the ring implantation remains standard practice, the choice of ring type and configuration is still a matter of ongoing debate.

Carpentier first described the importance of a “remodelling annuloplasty” with a rigid ring that mimics the mitral annulus in systole. Rigid rings are crucial for the treatment of annular dilatation, which is a consequence of chronic Primary Mitral Regurgitation (PMR). However, increased understanding of the pathophysiology of PMR enabled repair with complete semi-rigid rings, open rings or bands, which yield comparable results in terms of survival, freedom from reoperation and freedom from recurrent MR up to 14 years after surgery.^35^^,^^37–39^ Theoretically, open rings or bands in addition to posterior annulus stabilization, might have some technical and pathophysiological advantages. Compared with complete rings, posterior annuloplasty devices do not require suturing the anterior annulus, therefore resulting in a shorter, easier (especially when visibility of the anterior annulus is limited) and safer (lower risk of aortic valve injury) procedure. Moreover, bands are technically forgiving (lower risk of valve distortion) and, through repositioning of commissural stitches, they confer the possibility of readjustment (**Figure S2**).

From a pathophysiological standpoint, the implantation of a posterior annuloplasty device helps in preserving annular dynamics in diastole and systole, leaving larger diastolic opening and decreasing the risk of SAM. During systole, both the anterior leaflet and the anterior annulus move posteriorly and apically to allow expansion of the left ventricular outflow tract and the aortic root.^40^ As shown with 3D cine phase-contrast flow CMR semi-rigid complete rings restrict aortic annulus motion, disturbing aortic outflow patterns, while partial flexible or open rings and bands do not.^41^ Moreover, posterior partial annuloplasty has been associated with lower postoperative gradients when compared with complete rings,^26^^,^^42^ and this difference is even more evident during exercise.^43^

Although annuloplasty has been shown to abolish MAD,^10^^,^^44^ a proper comparison between the different types of rings in terms of efficacy in MAD elimination has not been performed yet. The area of maximal disjunction is located at the infero-lateral part of the posterior annulus (corresponding to P1-P2 leaflet segment), therefore making a posterior annuloplasty device potentially enough to ensure MAD elimination after mitral repair. Anyhow, careful attention is required in this context when placing the ring stitches, as rare cases of post-operative MAD persistence have been reported.^10^

## Special conditions

### Syndromic MVP

Syndromic MVP has been associated with several genetic conditions with high genotypic and phenotypic heterogeneity (**Table S1**). The biomedical pathways associated with it include altered migration of primordial cell populations, increased TGF-beta signalling, impaired synthesis and remodelling of extracellular matrix (ECM) components, as well as alterations in proteoglycan production. Most of them result in an increased proliferation of valve interstitial cells as well as migration of inflammatory cells into the leaflet tissue.^45^ Furthermore, secretion of ECM proteins, accumulation of low stiffness collagen fibres and calcium deposition contribute to further activation of inflammatory pathways. This results in increased leaflet thickness and development of excessive tissue.^46^ In contrast to non-syndromic MVP, syndromic conditions are characterized by increased fibrosis and calcifications which further alter valve stiffness and geometry. Progressing tissue degeneration leads to increased mechanical stress on valvular and subvalvular structures, altered proteoglycan production and apoptosis which activates a vicious cycle leading to chordae rupture. The presence of mechanical stress causes accelerated progression of MR with an annual increase in regurgitant volume of 18.4 ml per year.^46^

MR represents, after aortic aneurysms, the second most common cardiovascular pathology of patients with Marfan syndrome. MVP, MAD and excessive valve tissue indicative of Barlow’s valve are typically found in Marfan patients with MV involvement. In the largest series to date, MV repair was associated with improved survival as compared to replacement. However, one-third of the patients had at least moderate recurrent MR at a median follow-up of 16 years, while only 2 patients underwent reoperation.^47^

### Mitral annular calcifications

MAC is a degenerative process of the fibrous portion of the atrioventricular junction found in 8%–15% of the general population and when present, is associated with significant MV disease. The calcification pattern starts at the posterior annulus and extends anteriorly towards the fibrotic trigones.^48^ This degenerative process is accelerated by mechanical stress induced by increased filling pressures (ie arterial hypertension, atrial fibrillation) or by annulus deformation (ie hypertrophic cardiomyopathy or MAD).^49^

**Figure 6.**
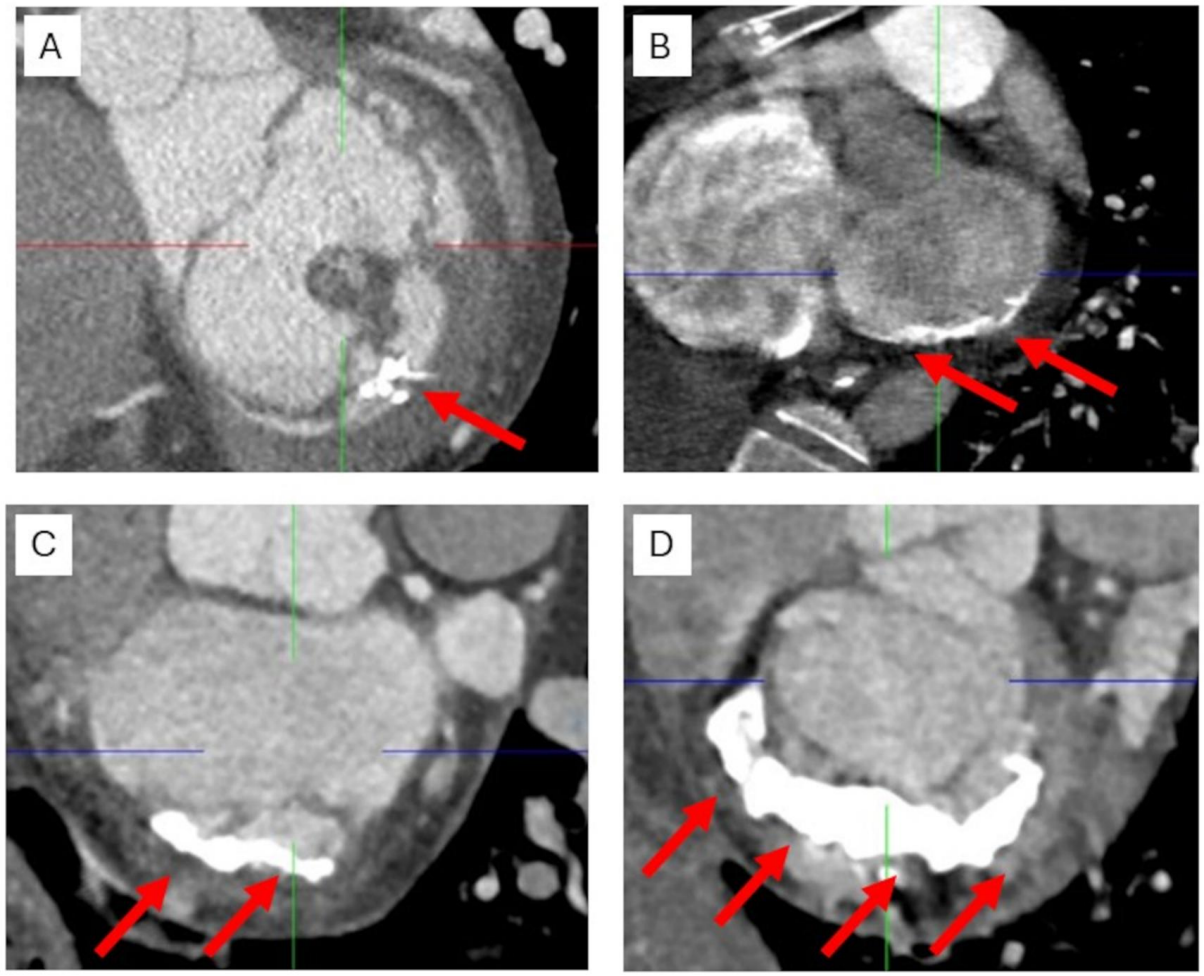
CT Scan from 4 Different Patients Presenting with Mitral Annular Calcification (Arrows) at Different Stages. Spotty calcification affecting a small part of the posterior annulus (A). Thin calcification affecting only the hinge of the posterior leaflet corresponding to P1 and P2 segment (B). Moderate calcification involving part of the posterior annulus and the base of the posterior leaflet (C). Heavy (Shoe-Horse-Like) calcification of the posterior annulus affecting part of the posterior leaflet (D).

From a pathophysiological view, the mitral annulus is exposed to significant mechanical stress which activates inflammation and subsequent fibrosis. The presence of myofibroblastic differentiation of valvular interstitial cells as well as infiltration and proliferation of inflammatory cells support the hypothesis of calcification through mechanotransduction.^50^ The hypermobile annular tissue gradually becomes fibrotic, whereas fibrosis may extend from the annulus to the LV myocardium and/or the papillary muscles.^51^ Calcification is the result of alterations in interactions between valve interstitial cells and ECM, which trigger further calcification, tissue degeneration, and chordal rupture, thus exacerbating MR. Patients may be referred to surgery or MV intervention at different stages of MAC (**Figure 6**).

Surgical techniques may vary according to the extension of MAC and may include standard types of repair without the need to remove calcium in patients with limited subannular calcifications. In more advanced cases, detachment of the posterior leaflet from the annulus followed by annular debridement with annulus reinforcement and reattachment of the posterior leaflet can be considered (ie young patients with extensive MAC). Annuloplasty sutures are more atrially placed in these cases to avoid rupture of the calcium bar. It is important to mention that the placement of an annuloplasty ring does not target annular hypermobility in cases of MAC but rather to re-establish an adequate coaptation area and avoid residual MR. Older patients usually require surgical or transcatheter MV replacement depending on clinical and anatomical conditions and the risk of atrioventricular dehiscence. Surgical MV replacement can be performed with annular debridement and patch reconstruction prior to prosthesis implantation or using a transcatheter heart valve with no or minimal decalcification.

### Systolic anterior motion

Systolic anterior motion (SAM) refers to a mechanical obstruction of the left ventricular outflow when dragging forces during systole push the anterior mitral leaflet towards the interventricular septum. Patients with excessive leaflet tissue referred for treatment of primary mitral disease are at risk of SAM, especially if conditions such as hypermobile anterior leaflet, hypertrophy of the interventricular septum, aortomitral angle <120°, C-sept distance < 25 mm, tall posterior leaflet and use of smaller annuloplasty rings are present.^52^ Patients with MAD referred for surgical treatment commonly present with a Barlow’s phenotype with excessive leaflet tissue and bi-leaflet curling and SAM can occur after repair if leaflet coaptation is anteriorly misplaced. Treatment of SAM can be achieved by improving ventricular filling, treating tachycardia and abandon inotropic stimulation. In patients with severe SAM accompanied by severe residual MR despite conservative measures, surgical re-repair is indicated. This can be achieved by the use of significantly larger annuloplasty rings, by ventricularization of the excessive posterior leaflet or by surgical edge-to-edge techniques.^53^

### Assessment of MAD treatment—long-term results

The assessment of long-term results after MAD treatment is based on multimodality imaging and electrocardiography (ECG) monitoring.

#### ECG-monitoring

Incompletely treated MAD after mitral annuloplasty may be related to persistent ventricular arrhythmia.^10^ Furthermore, even after successful MVR with accurate annuloplasty stabilization of MAD, worsening of arrhythmias might still happen in around 20% of patients after surgery.^44^ According to the European Heart Rhythm Association (EHRA) expert consensus statement, a standard 24-h Holter monitoring and a close follow-up are warranted in patients with MVP or complex ventricular ectopy, respectively.^9^ Additionally, due to the possibility of disease progression, periodic Holter may be advised as part of the routine follow-up. Developing specific risk stratification protocols is necessary for patients not responsive to the antiarrhythmic effect of MV surgery. Meticulous electrophysiologic evaluation is of utmost importance in these patients. Reasonable medical management options include beta blockers and amiodarone.

According to the EHRA expert consensus, an implantable loop recorder (ILR) may be advised in patients with MVP and unexplained syncope in whom non-invasive ECG monitoring remains inconclusive.^9^ ILR may also be advised to patients with arrhythmic MVP with negative findings on CMR and high-risk features (**Table 2**).

**Table 2.** Risk Features of Malignant Arrhythmias and Sudden Cardiac Death

High risk features	Phenotypic risk features
Sustained ventricular tachycardia Non-sustained ventricular tachycardia Unexplained syncope	T-wave inversion in the inferior leads Repetitive documented polymorphic premature ventricular contractions Mitral annular disjunction Redundant mitral leaflets Enlarged left atrium Ejection fraction ≤50%

Primary prevention with an ICD is recommended in patients with arrhythmic MVP, left ventricular ejection fraction <35%, and symptomatic heart failure despite ≥3 months of optimal medical therapy.^9^ An ICD may also be considered in patients with a history of unexplained syncope and sustained or non-sustained ventricular tachycardia likely arising from the mitral apparatus or in patients with one high-risk feature and 2 or more phenotypic risk features. Patients with arrhythmic MVP and a documented history of ventricular fibrillation or haemodynamically not tolerated ventricular tachycardia should receive an ICD as a secondary prevention strategy.

#### Echocardiography

Immediately after MV repair, MAD should no longer be detected on intraoperative transoesophageal echocardiography. Procedures performed at a more advanced stage of MAD, that is involving the anterior leaflet or both leaflets are associated with a higher risk of recurrent MR when compared to an isolated posterior leaflet prolapse.^54^^,^^55^ Therefore, baseline echocardiographic assessment following MV repair is essential to detect residual MR.^54^^,^^56^ The recurrence of significant MR is more common in the first year following repair.^54^ This allows an increase of the follow-up time interval if the postoperative result remains stable in the long term.^57^ Long-term follow-up should take into consideration serial assessment of function and dimension of the left ventricle.^54^ Early repair failure was found to have a different mechanism compared to late failure, is more likely to relate to a technical failure and is more amenable to redo repair.^58^ Late recurrent MR is typically related to the progression of the degenerative MV disease rather than failure of the repair^59^ (**Figure S3**).

## Impact of surgery on arrhythmias

The genesis of ventricular arrhythmias (VA) in patients with MAD is a multifactorial process. Several potential contributors are implicated, including severe MR,^60^ myocardial fibrosis,^61–64^ prolapse-induced myocardial stretch.^65^^,^^66^ MV surgery, by stabilizing the annulus, eliminating prolapse, and correcting MR, is theoretically expected to reduce electrical instability and VA. However, current evidence on its efficacy remains controversial.

Older case reports and series described the potentially beneficial role of MV surgery in alleviating symptoms and reducing VA burden in patients with MVP and severe arrhythmias but without significant MR.^67^ A renewed interest in the topic has recently fueled a surge of new investigations on the antiarrhythmic effects of surgery in patients with concomitant significant MR, but the results have been inconsistent. While some series reported a post-surgical reduction in VA burden, many others suggested that mitral surgery may not uniformly affect it.^68^^,^^69^ Undoubtedly, the presence of MAD is associated with a 3-fold increased long-term risk for ventricular arrhythmias as compared to non-MAD patients. This risk remains similar regardless of MV repair or replacement.^70^

As a matter of fact, the proportion of arrhythmogenic MVP patients free from recurrent VA at follow-up ranges between 55%^44^ and 75.9%,^71^ and different characteristics have been associated with persistent post-surgical VA. Those include age at intervention, complexity of pre-operative VA^71^ and the absence of posterior MAD, thus suggesting the presence of additional electrical triggers, which may not be addressed by mitral surgery.^44^^,^^72^ Moreover, no repair technique has yet demonstrated superior antiarrhythmic effects over the others.^44^^,^^71^ A potential explanation for the unpredictability of surgery’s impact on VA relies in MVP pathophysiology itself. While an early referral seems imperative to ensure antiarrhythmic efficacy, this premise clashes with the reality that MR usually develops late, after years of prolapse and stress on the left ventricular myocardium.^71^ Some groups have proposed adding concomitant cryoablation of the papillary muscles during MV surgery.^73^^,^^74^ It remains though unclear to which extent the induction of additional fibrotic lesions will decrease or increase the existing arrhythmic risk. Multimodality imaging, using late gadolinium enhancement CMR^75^ or hybrid PET/CMR,^76^ allows identifying areas of myocardial fibrosis which can be addressed during surgery.

It is worth noting that some reports^44^^,^^77^ have described the onset of a significant post-operative arrhythmic burden after successful surgery in a subset of patients without history of arrhythmia. This finding indicates the possibility of a myocardial excitability progression during MVP subjects’ natural history. A proarrhythmic effect of surgery itself cannot be excluded, and arrhythmogenic mechanisms—such as the creation of new substrate through post-surgical scarring—are postulated but have not been confirmed. A description of the available evidence on the impact of mitral surgery on VA is summarized in **Table S2**. At least 4 prospective trials (NCT05562804, NCT06255457, NCT05051033, and R01HL166720) are ongoing and will soon provide more insights on the topic.

## Gaps in evidence

Despite increasing recognition of mitral annular disjunction (MAD) as a clinically relevant entity several important gaps in evidence persist. First, the lack of standardized diagnostic criteria across imaging modalities leads to inconsistent identification and limits comparability between studies. Second, the natural history of MAD remains poorly defined. Longitudinal data are lacking to determine which patients with moderate MR and MAD are at greatest risk of progression to severe disease or arrhythmic complications. Consequently, the timing of surgical intervention in these patients remains uncertain. Current guidelines largely reflect the presence of severe MR and do not incorporate MAD-specific biomechanics or arrhythmic risk. Furthermore, although MV repair can anatomically correct MAD, its influence on ventricular arrhythmia burden is inconsistently reported. The pathophysiological mechanisms linking MAD and arrhythmogenesis also remain incompletely understood, with ongoing debate as to whether MAD constitutes a true arrhythmic substrate or reflects broader myocardial vulnerability due to local fibrosis. In addition, the role of MAC in the context of MAD is insufficiently studied. The causative relationship between annular hypermobility and calcification remains speculative, and surgical outcomes in patients with coexisting MAC are under-reported. Finally, there are no validated risk stratification tools for the postoperative assessment of arrhythmic risk or repair durability, and the genetic and paediatric aspects of MAD remain largely unexplored. These gaps limit tailored decision-making and underscore the need for prospective, multimodal research.

## Supplementary Material

ezaf461_Supplementary_Data

## Data Availability

The data underlying this article are available in the article and in its online supplementary material.

## References

[1] Angelini A, Ho SY, Anderson RH, Davies MJ, Becker AE. A histological study of the atrioventricular junction in hearts with normal and prolapsed leaflets of the mitral valve. Br Heart J. 1988;59:712-716. 3395530 10.1136/hrt.59.6.712 PMC1276881

[2] Wunderlich NC, Ho SY, Flint N, Siegel RJ. Myxomatous mitral valve disease with mitral valve prolapse and mitral annular disjunction: clinical and functional significance of the coincidence. J Cardiovasc Dev Dis. 2021;8:9. 33498935 10.3390/jcdd8020009 PMC7911536

[3] Hutchins GM, Moore GW, Skoog DK. The association of floppy mitral valve with disjunction of the mitral annulus fibrosus. N Engl J Med. 1986;314:535-540. 3945291 10.1056/NEJM198602273140902

[4] Toh H, Mori S, Izawa Y, et al Prevalence and extent of mitral annular disjunction in structurally normal hearts: comprehensive 3D analysis using cardiac computed tomography. Eur Heart J Cardiovasc Imaging. 2021;22:614-622. 33713105 10.1093/ehjci/jeab022

[5] Fiore G, Rizza V, Ingallina G, et al Prevalence of diastolic and systolic mitral annular disjunction in patients with mitral valve prolapse. J Am Soc Echocardiogr. 2025;38:1-11. 39442734 10.1016/j.echo.2024.10.004

[6] Hiemstra YL, Tomsic A, Gripari P, et al Evolution from mitral annular dysfunction to severe mitral regurgitation in Barlow’s disease. Interact CardioVasc Thorac Surg. 2021;32:506-514. 33367628 10.1093/icvts/ivaa304 PMC8906724

[7] Klautz RJ, Tomšič A, Palmen M, van Brakel TJ, Perier P. Optimal surgical mitral valve repair in Barlow’s disease: the concept of functional prolapse. Multimed Man Cardiothorac Surg. 2017;2016. 10.1510/mmcts.2016.001 28106961

[8] Amano M, Izumi C, Toki M, et al Mitral valve early systolic billowing induces following annular expansion and leaflet augmentation in Barlow’s disease: sequential analysis using 3D echocardiography. Eur Heart J Cardiovasc Imaging. 2024;25:784-794. 38289248 10.1093/ehjci/jeae031

[9] Sabbag A, Essayagh B, Barrera JDR, et al EHRA expert consensus statement on arrhythmic mitral valve prolapse and mitral annular disjunction complex in collaboration with the ESC Council on valvular heart disease and the European association of cardiovascular imaging endorsed by the Heart Rhythm Society, by the Asia Pacific Heart Rhythm Society, and by the Latin American Heart Rhythm Society. Europace. 2022;24:1981-2003. 35951656 10.1093/europace/euac125 PMC11636573

[10] Essayagh B, Mantovani F, Benfari G, et al Mitral annular disjunction of degenerative mitral regurgitation: three-dimensional evaluation and implications for mitral repair. J Am Soc Echocardiogr. 2022;35:165-175. 34517112 10.1016/j.echo.2021.09.004

[11] Pavon AG, Arangalage D, Pascale P, et al Myocardial extracellular volume by T1 mapping: a new marker of arrhythmia in mitral valve prolapse. J Cardiovasc Magn Reson. 2021;23:102. 34517908 10.1186/s12968-021-00797-2 PMC8438990

[12] Dejgaard LA, Skjølsvik ET, Lie ØH, et al The mitral annulus disjunction arrhythmic syndrome. J Am Coll Cardiol. 2018;72:1600-1609. 30261961 10.1016/j.jacc.2018.07.070

[13] Kuetting DLR, Dabir D, Luetkens J, et al Flip angle optimization for balanced SSFP: cardiac cine imaging following the application of standard extracellular contrast agent (gadobutrol). J Magn Reson Imaging. 2018;47:255-261. 28429574 10.1002/jmri.25728

[14] Zugwitz D, Fung K, Aung N, et al Mitral annular disjunction assessed using CMR imaging: insights from the UK Biobank population study. JACC Cardiovasc Imaging. 2022;15:1856-1866. 36280553 10.1016/j.jcmg.2022.07.015 PMC9640354

[15] Figliozzi S, Georgiopoulos G, Lopes PM, et al Myocardial fibrosis at cardiac MRI helps predict adverse clinical outcome in patients with mitral valve prolapse. Radiology. 2023;306:112-121. 36098639 10.1148/radiol.220454

[16] Kukavica D, Guglielmo M, Baggiano A, et al Arrhythmic mitral valve prolapse: introducing an era of multimodality imaging-based diagnosis and risk stratification. Diagnostics (Basel). 2021;11:467 33800155 10.3390/diagnostics11030467 PMC7999774

[17] Praz F, Borger MA, Lanz J, ESC/EACTS Scientific Document Group, et al 2025 ESC/EACTS guidelines for the management of valvular heart disease: developed by the task force for the management of valvular heart disease of the European Society of Cardiology (ESC) and the European Association for Cardio-Thoracic Surgery (EACTS). Eur J Cardiothorac Surg. 2025;67:ezaf276.40878291

[18] Gillinov AM, Cosgrove DM, Wahi S, et al Is anterior leaflet repair always necessary in repair of bileaflet mitral valve prolapse? Ann Thorac Surg. 1999;68:820-823; discussion 824. 10509968 10.1016/s0003-4975(99)00805-x

[19] Ashikhmina E, Schaff HV, Daly RC, et al Risk factors and progression of systolic anterior motion after mitral valve repair. J Thorac Cardiovasc Surg. 2021;162:567-577. 32173099 10.1016/j.jtcvs.2019.12.106

[20] Asai T, Kinoshita T, Nishimura O, Kambara A, Suzuki T, Matsubayashi K. A novel design of posterior leaflet butterfly resection for mitral valve repair. Innovations (Phila). 2011;6:54-56. 22437805 10.1097/IMI.0b013e31820c0107

[21] Cevasco M, Myers PO, Elbardissi AW, Cohn LH. Foldoplasty: a new and simplified technique for mitral valve repair that produces excellent medium-term outcomes. Ann Thorac Surg. 2011;92:1634-1637; discussion 1637-1638. 22051259 10.1016/j.athoracsur.2011.05.123

[22] Perier P, Hohenberger W, Lakew F, et al Toward a new paradigm for the reconstruction of posterior leaflet prolapse: midterm results of the “respect rather than resect” approach. Ann Thorac Surg. 2008;86:718-725; discussion 718-25. 18721552 10.1016/j.athoracsur.2008.05.015

[23] Perier P. A case of mitral annulus disjunction repaired with the “snail” technique. JTCVS Tech. 2023;22:92-93. 38152180 10.1016/j.xjtc.2023.09.009 PMC10750851

[24] De Bonis M, Lapenna E, Del Forno B, et al Minimally invasive or conventional edge-to-edge repair for severe mitral regurgitation due to bileaflet prolapse in Barlow’s disease: does the surgical approach have an impact on the long-term results? Eur J Cardiothorac Surg. 2017;52:131-136. 28407104 10.1093/ejcts/ezx032

[25] Pölzl L, Gollmann-Tepeköylü C, Nägele F, et al Five-year outcomes of different techniques for minimally invasive mitral valve repair in Barlow’s disease. Eur J Cardiothorac Surg. 2024;65:ezae213. 38781502 10.1093/ejcts/ezae213 PMC11150856

[26] Tomšič A, Sandoval E, Meucci MC, et al The impact of annuloplasty ring or band implantation on post-repair mitral valve haemodynamic performance. Eur J Cardiothorac Surg. 2023;64:ezad307. 37688566 10.1093/ejcts/ezad307 PMC10517645

[27] Del Forno B, Tavana K, Ruffo C, et al Neochordae implantation versus leaflet resection in mitral valve posterior leaflet prolapse and dilated left ventricle: a propensity score matching comparison with long-term follow-up. Eur J Cardiothorac Surg. 2023;64: ezad274. 37551944 10.1093/ejcts/ezad274 PMC10693437

[28] van Wijngaarden AL, Tomšič A, Mertens BJA, et al Mitral valve repair for isolated posterior mitral valve leaflet prolapse: the effect of respect and resect techniques on left ventricular function. J Thorac Cardiovasc Surg. 2022;164:1488-1497.e3. 33744010 10.1016/j.jtcvs.2021.02.017

[29] Nisivaco S, McCarthy PM, Kruse J, Andre AC, Zhao M, Thomas JD. Late results of chord transfer and other techniques for anterior leaflet repair without neochords. J Thorac Cardiovasc Surg. 2024;168:1045-1056.e3. 37453720 10.1016/j.jtcvs.2023.05.047

[30] Brescia AA, Watt TMF, Rosenbloom LM, et al Michigan Mitral Research Group. Anterior versus posterior leaflet mitral valve repair: a propensity-matched analysis. J Thorac Cardiovasc Surg. 2021;162:1087-1096.e3. 32305185 10.1016/j.jtcvs.2019.11.148 PMC7483316

[31] David TE, David CM, Lafreniere-Roula M, Manlhiot C. Long-term outcomes of chordal replacement with expanded polytetrafluoroethylene sutures to repair mitral leaflet prolapse. J Thorac Cardiovasc Surg. 2020;160:385-394.e1. 31570218 10.1016/j.jtcvs.2019.08.006

[32] Kawamoto N, Fujita T, Fukushima S, et al Should annuloplasty prosthesis be selected dependent on the location of prolapse in mitral valve repair for type II dysfunction? J Thorac Cardiovasc Surg. 2017;154:1915-1924.e6. 28755880 10.1016/j.jtcvs.2017.06.049

[33] Alfieri O, Maisano F, De Bonis M, et al The double-orifice technique in mitral valve repair: a simple solution for complex problems. J Thorac Cardiovasc Surg. 2001;122:674-681. 11581597 10.1067/mtc.2001.117277

[34] Maisano F, Caldarola A, Blasio A, De Bonis M, La Canna G, Alfieri O. Midterm results of edge-to-edge mitral valve repair without annuloplasty. J Thorac Cardiovasc Surg. 2003;126:1987-1997. 14688717 10.1016/s0022-5223(03)01291-1

[35] Del Forno B, Carino D, Bisogno A, et al Mitral repair with complete rings or posterior bands in Barlow disease: long-term results. Ann Thorac Surg. 2023;115:421-427. 35780815 10.1016/j.athoracsur.2022.06.015

[36] De Bonis M, Lapenna E, Taramasso M, et al Very long-term durability of the edge-to-edge repair for isolated anterior mitral leaflet prolapse: up to 21 years of clinical and echocardiographic results. J Thorac Cardiovasc Surg. 2014;148:2027-2032. 24755329 10.1016/j.jtcvs.2014.03.041

[37] David TE, David CM, Manlhiot C. Simplici-T annuloplasty band for mitral valve repair for degenerative disease. Ann Thorac Surg. 2014;98:1551-1556. 25201724 10.1016/j.athoracsur.2014.06.016

[38] Khamooshian A, Buijsrogge MP, de Heer F, Gründeman PF. Mitral valve annuloplasty rings: review of literature and comparison of functional outcome and ventricular dimensions. Innovations (Phila). 2014;9:399-415. 25469460 10.1177/155698451400900603

[39] Baccelli A, Lapenna E, Del Forno B, et al Long-term results of mitral repair with complete semi-rigid rings vs posterior flexible bands. Ann Thorac Surg. 2021;112:756-761. 33275928 10.1016/j.athoracsur.2020.11.006

[40] James L, Grossi EA, Loulmet DF, Galloway AC. Semirigid posterior annuloplasty band: reshaping the mitral orifice while preserving its physiology. JTCVS Tech. 2021;10:37-42. 34977703 10.1016/j.xjtc.2021.10.001 PMC8691863

[41] Morichi H, Itatani K, Yamazaki S, et al Influences of mitral annuloplasty on left ventricular flow dynamics assessed with 3-dimensional cine phase-contrast flow magnetic resonance imaging. J Thorac Cardiovasc Surg. 2022;163:947-959. 32690416 10.1016/j.jtcvs.2020.04.127

[42] Kakuta T, Fukushima S, Minami K, et al What is the optimal mitral valve repair for isolated posterior leaflet prolapse to achieve long-term durability? J Am Heart Assoc. 2023;12:e028607. 37232245 10.1161/JAHA.122.028607 PMC10382008

[43] Mesana TG, Lam BK, Chan V, Chen K, Ruel M, Chan K. Clinical evaluation of functional mitral stenosis after mitral valve repair for degenerative disease: potential affect on surgical strategy. J Thorac Cardiovasc Surg. 2013;146:1418-1423; discussion 1423-1425. 24075470 10.1016/j.jtcvs.2013.08.011

[44] Ascione G, Azzola Guicciardi N, Lorusso R, et al The impact of mitral valve surgery on ventricular arrhythmias in patients with Barlow’s disease: preliminary results of a prospective study. Interdiscip Cardiovasc Thorac Surg. 2023;36:ivad073. 10.1093/icvts/ivad073 PMC10212537 37166486

[45] van Wijngaarden AL, Kruithof BPT, Vinella T, Barge-Schaapveld D, Ajmone Marsan N. Characterization of degenerative mitral valve disease: differences between fibroelastic deficiency and Barlow’s disease. J Cardiovasc Dev Dis. 2021;8:23. 33671724 10.3390/jcdd8020023 PMC7926852

[46] Ronco D, Buttiglione G, Garatti A, Parolari A. Biology of mitral valve prolapse: from general mechanisms to advanced molecular patterns-a narrative review. Front Cardiovasc Med. 2023;10:1128195. 37332582 10.3389/fcvm.2023.1128195 PMC10272793

[47] David TE, Park J, Steve Fan CP. Mitral valve surgery in patients with Marfan syndrome. J Thorac Cardiovasc Surg. 2025;169:599-605. 38678476 10.1016/j.jtcvs.2024.01.046

[48] Chehab O, Roberts-Thomson R, Bivona A, et al Management of patients with severe mitral annular calcification: JACC state-of-the-art review. J Am Coll Cardiol. 2022;80:722-738. 35953138 10.1016/j.jacc.2022.06.009

[49] Abramowitz Y, Jilaihawi H, Chakravarty T, Mack MJ, Makkar RR. Mitral annulus calcification. J Am Coll Cardiol. 2015;66:1934-1941. 26493666 10.1016/j.jacc.2015.08.872

[50] Pagnozzi LA, Butcher JT. Mechanotransduction mechanisms in mitral valve physiology and disease pathogenesis. Front Cardiovasc Med. 2017;4:83. 29312958 10.3389/fcvm.2017.00083 PMC5744129

[51] Willner N, Burwash IG, Beauchesne L, et al Natural history of mitral annular calcification and calcific mitral valve disease. J Am Soc Echocardiogr. 2022;35:925-932. 35618253 10.1016/j.echo.2022.05.007

[52] Varghese R, Itagaki S, Anyanwu AC, Trigo P, Fischer G, Adams DH. Predicting systolic anterior motion after mitral valve reconstruction: using intraoperative transoesophageal echocardiography to identify those at greatest risk. Eur J Cardiothorac Surg. 2014;45:132-137; discussion 137-138. 23657548 10.1093/ejcts/ezt234

[53] Alfieri O, Lapenna E. Systolic anterior motion after mitral valve repair: where do we stand in 2015? Eur J Cardiothorac Surg. 2015;48:344-346. 26142473 10.1093/ejcts/ezv230

[54] Suri RM, Clavel M-A, Schaff HV, et al Effect of recurrent mitral regurgitation following degenerative mitral valve repair: long-term analysis of competing outcomes. J Am Coll Cardiol. 2016;67:488-498. 26846946 10.1016/j.jacc.2015.10.098

[55] David TE, Armstrong S, McCrindle BW, Manlhiot C. Late outcomes of mitral valve repair for mitral regurgitation due to degenerative disease. Circulation. 2013;127:1485-1492. 23459614 10.1161/CIRCULATIONAHA.112.000699

[56] Jouan J, Berrebi A, Chauvaud S, Menasché P, Carpentier A, Fabiani JN. Mitral valve reconstruction in Barlow disease: long-term echographic results and implications for surgical management. J Thorac Cardiovasc Surg. 2012;143:S17-20. 22154786 10.1016/j.jtcvs.2011.11.016

[57] Tomšic A, Hiemstra YL, van der Pas SL, et al Early and long-term outcomes of mitral valve repair for Barlow’s disease: a single-centre 16-year experience. Interact CardioVasc Thorac Surg. 2018;26:783-789. 29340624 10.1093/icvts/ivx423

[58] Moore RA, Wierup P, Tappuni S, et al Reoperation after early and late failure of mitral valve repair for degenerative disease. J Thorac Cardiovasc Surg. 2024;167:1251-1262.e8. 36323616 10.1016/j.jtcvs.2022.09.033

[59] Suri RM, Schaff HV, Dearani JA, et al Recurrent mitral regurgitation after repair: should the mitral valve be re-repaired? J Thorac Cardiovasc Surg. 2006;132:1390-1397. 17140963 10.1016/j.jtcvs.2006.07.018

[60] Grigioni F, Enriquez-Sarano M, Ling LH, et al Sudden death in mitral regurgitation due to flail leaflet. J Am Coll Cardiol. 1999;34:2078-2085. 10588227 10.1016/s0735-1097(99)00474-x

[61] Dieterlen M-T, Klaeske K, Spampinato R, et al Histopathological insights into mitral valve prolapse-induced fibrosis. Front Cardiovasc Med. 2023;10:1057986. 36960475 10.3389/fcvm.2023.1057986 PMC10028262

[62] Bui AH, Roujol S, Foppa M, et al Diffuse myocardial fibrosis in patients with mitral valve prolapse and ventricular arrhythmia. Heart. 2017;103:204-209. 27515954 10.1136/heartjnl-2016-309303 PMC5237392

[63] Basso C, Perazzolo Marra M, Rizzo S, et al Arrhythmic mitral valve prolapse and sudden cardiac death. Circulation. 2015;132:556-566. 26160859 10.1161/CIRCULATIONAHA.115.016291

[64] Nagata Y, Bertrand PB, Baliyan V, et al Abnormal mechanics relate to myocardial fibrosis and ventricular arrhythmias in patients with mitral valve prolapse. Circ Cardiovasc Imaging. 2023;16:e014963. 37071717 10.1161/CIRCIMAGING.122.014963 PMC10108844

[65] Malyshev Y, Miller MA. Catheter ablation of arrhythmic mitral valve prolapse: should we be MAD? no, quite the opposite. JACC Clin Electrophysiol. 2023;9:1276-1278. 37354189 10.1016/j.jacep.2023.05.008

[66] Park MH, van Kampen A, Melnitchouk S, et al Native and post-repair residual mitral valve prolapse increases forces exerted on the papillary muscles: a possible mechanism for localized fibrosis? Circ Cardiovasc Interv. 2022;15:e011928. 36538583 10.1161/CIRCINTERVENTIONS.122.011928 PMC9782735

[67] Pocock WA, Barlow JB, Marcus RH, Barlow CW. Mitral valvuloplasty for life-threatening ventricular arrhythmias in mitral valve prolapse. Am Heart J. 1991;121:199-202. 1985363 10.1016/0002-8703(91)90976-o

[68] Vaidya VR, DeSimone CV, Damle N, et al Reduction in malignant ventricular arrhythmia and appropriate shocks following surgical correction of bileaflet mitral valve prolapse. J Interv Card Electrophysiol. 2016;46:137-143. 26768434 10.1007/s10840-015-0090-5 PMC4925199

[69] Essayagh B, Sabbag A, Antoine C, et al The mitral annular disjunction of mitral valve prolapse: presentation and outcome. JACC Cardiovasc Imaging. 2021;14:2073-2087. 34147457 10.1016/j.jcmg.2021.04.029

[70] Lodin K, Da Silva CO, Wang Gottlieb A, et al Mitral annular disjunction and mitral valve prolapse: long-term risk of ventricular arrhythmias after surgery. Eur Heart J. 2025;46:2795-2805. 40230055 10.1093/eurheartj/ehaf195 PMC12277880

[71] Pandis D, David N, Ei-Eshmawi A, et al Noncomplex ventricular arrhythmia associated with greater freedom from recurrent ectopy at 1 year after mitral repair surgery. JTCVS Open. 2024;19:94-113. 39015439 10.1016/j.xjon.2024.04.005 PMC11247206

[72] Azzola Guicciardi N, Ascione G, Alfieri O, Maisano F, De Bonis M. When annuloplasty is not enough: a case report of ventricular arrhythmias stepwise abolition after mitral valve re-repair. Eur Heart J Case Rep. 2024;8:ytae305. 39006214 10.1093/ehjcr/ytae305 PMC11245692

[73] Vohra J, Morton JB, Morgan J, Tatoulis J. Cryoablation of papillary muscles at surgery for malignant ventricular arrhythmias due to mitral valve prolapse. Heart Lung Circ. 2022;31:1285-1290. 35697646 10.1016/j.hlc.2022.04.058

[74] El-Eshmawi A, Pandis D, Miller MA, et al Surgical cryoablation of papillary muscle PVCs during mitral valve surgery: therapeutic consideration for malignant MVP. J Am Coll Cardiol. 2020;76:3061-3062. 33334428 10.1016/j.jacc.2020.10.037

[75] Van De Heyning CM, Holtackers RJ, Nazir MS, et al Dark-blood late gadolinium enhancement CMR improves detection of papillary muscle fibrosis in patients with mitral valve prolapse. Eur J Radiol. 2022;147:110118. 34972057 10.1016/j.ejrad.2021.110118

[76] Miller MA, Devesa A, Robson PM, et al Arrhythmic mitral valve prolapse with only mild or moderate mitral regurgitation: characterization of myocardial substrate. JACC Clin Electrophysiol. 2023;9:1709-1716. 37227360 10.1016/j.jacep.2023.04.011 PMC13123559

[77] Tampakis K, Polytarchou K, Andrikopoulos G. New-onset ventricular arrhythmias after surgery for mitral valve prolapse: how to classify and manage? Europace. 2023;25:776-777. 10.1093/europace/euac207 PMC9934986 36413623

