# Supplementary material for: Mitral Annular Disjunction: A Roadmap for the Surgeon
Source: Eur J Cardiothorac Surg. 2025 Dec 17;68(1):ezaf461. doi: 10.1093/ejcts/ezaf461 (PMC12957924; doi:10.1093/ejcts/ezaf461)
Supplement: ezaf461_Supplementary_Data [file ezaf461_Supplementary_Data.zip › Supplementary tables and legends of supplementary figures.docx]

**Supplementary Tables**

**Abbreviations**

AMVD Arrhythmogenic mitral valve disease

CMR Cardiac magnetic resonance

CT Computed tomography

ECG Electrocardiography

ECM Extracellular matrix

ICD Implantable cardiac defibrillator

LGE Late gadolinium enhancement

LVEF Left ventricular ejection fraction

LVESD Left ventricular end-systolic diameter

LVOT Left ventricular outflow tract

MAC Mitral annular calcifications

MAD Mitral annular disjunction

MPV Mitral valve prolapse

MV Mitral valve

SAM Systolic anterior motion

TTE Transthoracic echocardiography

VA Ventricular arrhythmias

**Table S1. Syndromic mitral prolapse: responsible genes and pathways leading to mitral regurgitation**

| **Condition** | **Responsible Gene** | **Mechanism** | **Median Prevalence of MVP** |
| --- | --- | --- | --- |
| Marfan | Fibrillin-1 | Upregulation of TGF-beta signaling | 56.7% |
| Loeys-Dietz | TGF-beta receptor 1 and 2 | Upregulation of TGF-beta signaling | 25% |
| Ehlers-Danlos | Collagen type 1 alpha 2 | Decreased stiffness of extracellular matrix, increased production of glycosaminoglycans | 6.2% |
| Ebstein Anomaly | Unknown | Unknown | 11.4% |
| Familial myxomatous valvular degeneration | Filamin A | Serotoninergic activation -> carcinoid-similar reaction | 38.1% |
| Fragile X | Fragile X Messenger Ribonucleoprotein regulator 1 | Unknown | 37.8% |
| Osteogenesis imperfecta | Procollagen subunits | Unknown | 5.4% |
| Pseudoxanthoma Elasticum | Unknown | Unknown | 43.4% |
| Down | Trisomy 21 | Decreased stiffness of extracellular matrix | 31.4% |
| Williams Beuren | Elastin | Elastin deficiency, upregulation of TGF-beta signaling | 22.3% |

**Supplementary Table S2.** Summary of the studies investigating the effects of mitral surgery on arrhythmias

| **Study** | **Number of patients** | **Study design** | **MVr vs MVR** | **Rhythm assessment technique** | **Rhythm assessment timeframe** | **Effects of surgery on arrhythmias** |
| --- | --- | --- | --- | --- | --- | --- |
| Reece et al. | 37 pts with Barlow’s syndrome (bi-leaflet MVP and chest pain, dyspnea or palpitations) and less than severe MR. 17 pts (46%) with VA | Retrospective | MVr (annuloplasty ± leaflet plication/chordal shortening) | Not disclosed | Not disclosed. Mean follow-up 4.7 years | 60% of pts had relief of one or more symptoms after surgery. No major VA was registered at follow-up. |
| Al-Bassam et al. | 2 pts with Barlow’s disease and less than severe MR but severe VA, not responsive to medications | Case series | MVr (posterior annuloplasty) | 24 hours Holter monitoring | 1 Holter before surgery, one at 3-month follow-up | Post-operative abolition of VA in both cases |
| Kay et al. | 6 pts with bi-leaflet prolapse and VA but no MR | Case series | MVr (suturing of prolapsing leaflets edges to the papillary muscles; annular narrowing via commissural stitches) | Holter monitoring | 1 Holter before surgery, at least one after surgery | Post-operative reduction in the arrhythmic burden in all the patients |
| Vohra et al. | 2 pts with MVP and malignant VA, but no significant MR | Case series | One MVR, one MVr (quadrangular resection + annuloplasty) | 24 hours Holter monitoring. EPS before and after surgery | Not disclosed. Mean follow-up 2.5 years | EPS performed 3 months after surgery still showed inducible VT in both patients |
| Naksuk et al. | 32 pts with bi-leaflet MVP | Retrospective | 30 (93.8%) MVr,  2 (6.2%) MVR | 24 hours Holter monitoring | Median time 98 days before and 102 days after surgery | No changes in pre- vs post-operative arrhythmic burden in the overall cohort.  A slight reduction of PVB frequency (>10%)  was recorded in younger patients (<60 years old) |
| Vaidya et al. | 5 pts with bi-leaflet MVP and pre-operative ICD implantation | Retrospective | 4 (80%) MVr,  1 (20%) MVR | ICD interrogation | Mean time 4.6 ± 2.9 years before versus 6.6 ± 4.2 years after surgery | After surgery, there was a reduction in VF, VT and ICD shocks incidence in all the patients |
| Essayagh et al. | 183 pts with isolated MVP | Retrospective | 170 (93%) MVr,  13 (7%) MVR | 24-hours Holter monitoring (median 1 [1-2] Holter per patient) | Not disclosed. Total clinical follow-up: 10.3 ± 3.0 years | Surgery was associated with a reduction of MAD-related excess of long-term  arrhythmic events, when compared with medical manage-  ment. |
| Ascione et al. | 63 patients with Barlow’s disease | Prospective | MVr | 24-hours Holter monitoring | 1 Holter before surgery, 1 holter at 3-month follow-up | 32% of the cohort had a significant arrhythmic burden at baseline. Surgery abolished VA in 55% of the cases. 18.6% of patients non-arrhythmic at baseline showed significant arrhythmias at follow-up |
| Pandis et al. | 62 pts with AMVP (defined as MVP + Lown grade ≥2 VA) | Retrospective | MVr | 24-hours holter/ICD interrogation (40% of pts).  Serial post-operative ECGs and presence/absence of palpitations (60% of pts) | Not disclosed. The median follow-up was 11.2 months (IQR, 1.6-30.7) | The 30-day and 1-year freedom from recurrent VA were 98.4% and 75.9%. Complex baseline VA (Lown grade ≥3) was the strongest predictor of recurrent VA |
| Brunec-Keller et al. | 82 pts with Barlow’s disease | Retrospective | MVr (52 % ring only, 48% ring + neochordae) | ECG (33% of pts) or 24 hours ECG Holter/ICD monitoring (67% of pts) | Not disclosed. Mean follow-up was 3.1 years (0.2 to 14.2 years | Surgery does not reduce VA burden in Barlow’s patients. Bi-leaflet MVP identified as an independent risk factor for increased VA burden during follow-up |
| Lodin et al | 599 patients with primary mitral disease, 96 pts with MAD | Retrospective | MVr (84%), MVR (16%) | ECG, ICD interrogation | Not disclosed. Mean Follow-up was 5.4 years (IQR: 2.8-7.5y) | Patients with MAD have a three-fold risk to present with ventricular arrhythmias after surgery, |

AMVP: Arrhythmic mitral valve prolapse; ECG: electrocardiogram; EPS: electrophysiological study; ICD: implantable cardioverter-defibrillator; MVP: mitral valve prolapse; MVr: mitral valve repair; MVR: mitral valve replacement; PVB: premature ventricular beats; VA: ventricular arrhythmias; VT: ventricular tachycardia; VF: ventricular fibrillation

**Supplementary Figures**

**Figure S1. Assessment of MAD by TOE.**

Pseudo-MAD (red arrows) as identified during systole **(A)** but not during diastole **(B)** from a mid-oesophageal long-axis view. True-MAD (double red arrows) as identified along the entire cardiac cycle, from systole to diastole **(C, D)**.

**Figure S2. A patient with MAD, mitral annular calcifications, severe MR, arrhythmogenic mitral valve prolapse and out of hospital cardiac arrest.**

Seventy seven years old male patient with arrhythmogenic mitral valve prolapse and MAD. **A.** Pre-op TTE showing a bi-leaflet mitral prolapse **B.** Late Gadolinium uptake showing fibrosis of the papillary muscle (arrow) **C.** Signs of mitral annular calcifications in coronary angiography (arrows).

**Figure S3. Repositioning of annuloplasty band/ring.** Ad-hoc repositioning of commissural stitches of a flexible band may be used as an adjunctive technique to increase leaflets coaptation in case of residual MR jets due to inadequate band sizing.

**Figure S4.** **Late mitral repair failure due to recurrent prolapse and progression of the disease.** Mitral regurgitation due bi-leaflet prolapse and MAD **(A)**. Recurrent mitral regurgitation late after mitral valve repair with neochordae and annuloplasty **(B)**. 3D-transesophageal echocardiography demonstrates residual or recurrent prolapse of the posterior leaflet at P2 and new flail at the medial commissure (arrows) **(C)**. 3D-transesophageal echocardiography surgical view with colour flow mapping demonstrates no regurgitation originating at P2, however severe regurgitation caused by the flail medial commissure **(D)**.
