# Supplementary figures and images for: Mitral Annular Disjunction: A Roadmap for the Surgeon
Source: Eur J Cardiothorac Surg. 2025 Dec 17;68(1):ezaf461. doi: 10.1093/ejcts/ezaf461 (PMC12957924; doi:10.1093/ejcts/ezaf461)

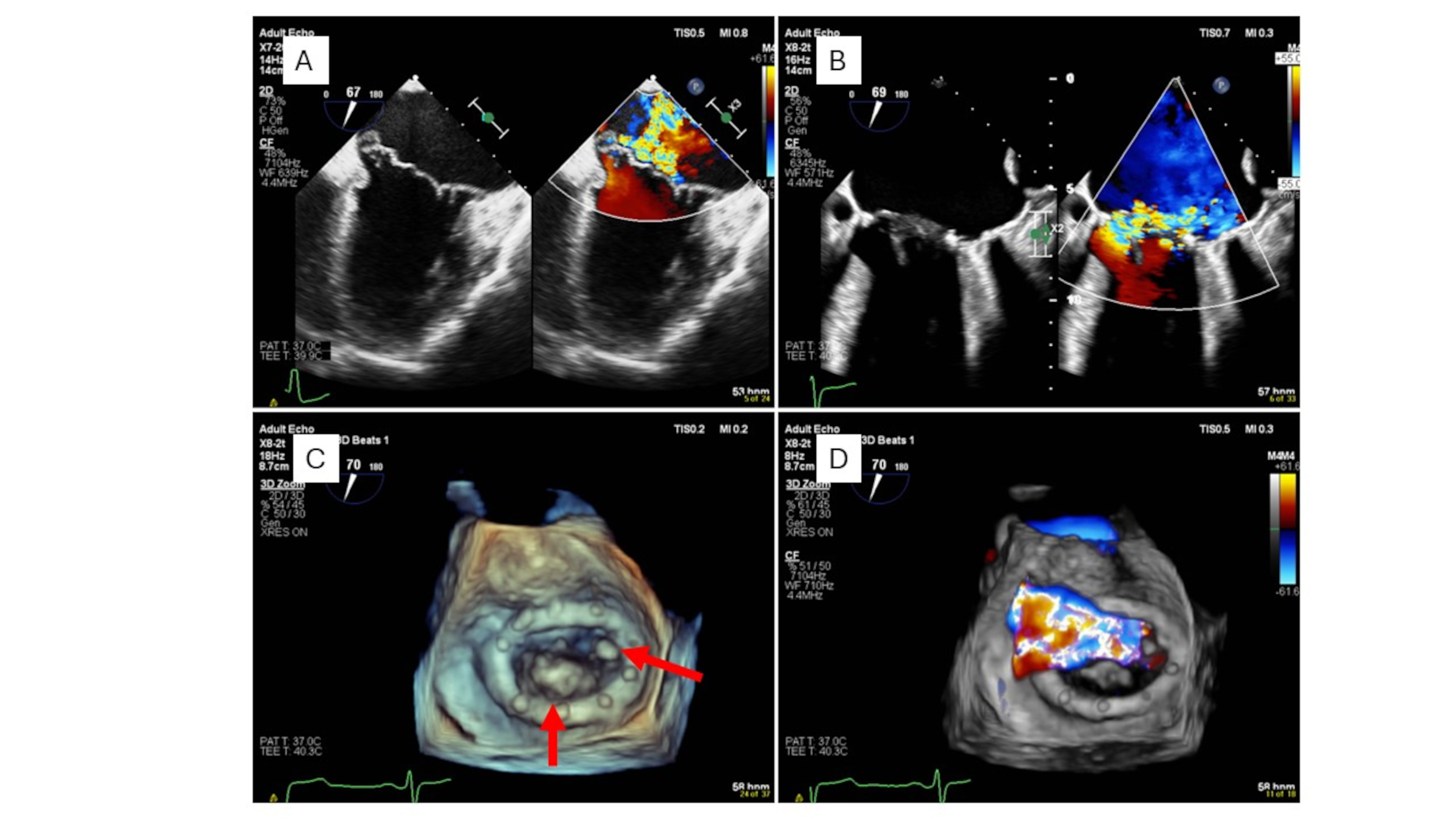

Supplement: ezaf461_Supplementary_Data [file ezaf461_Supplementary_Data.zip › Supplementary Figure 4 rev.JPG]

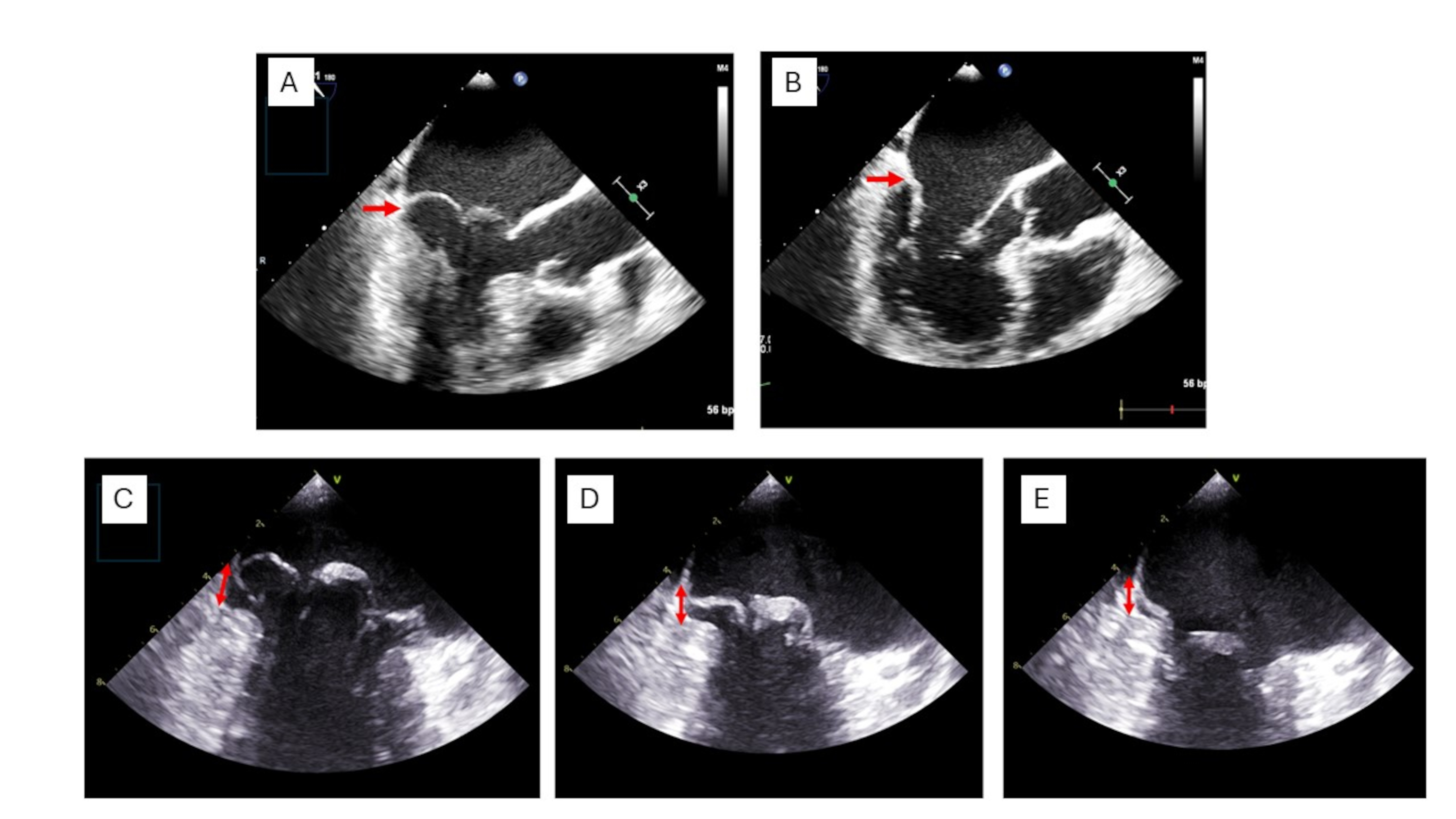

Supplement: ezaf461_Supplementary_Data [file ezaf461_Supplementary_Data.zip › Suppl Figure 1 rev.JPG]

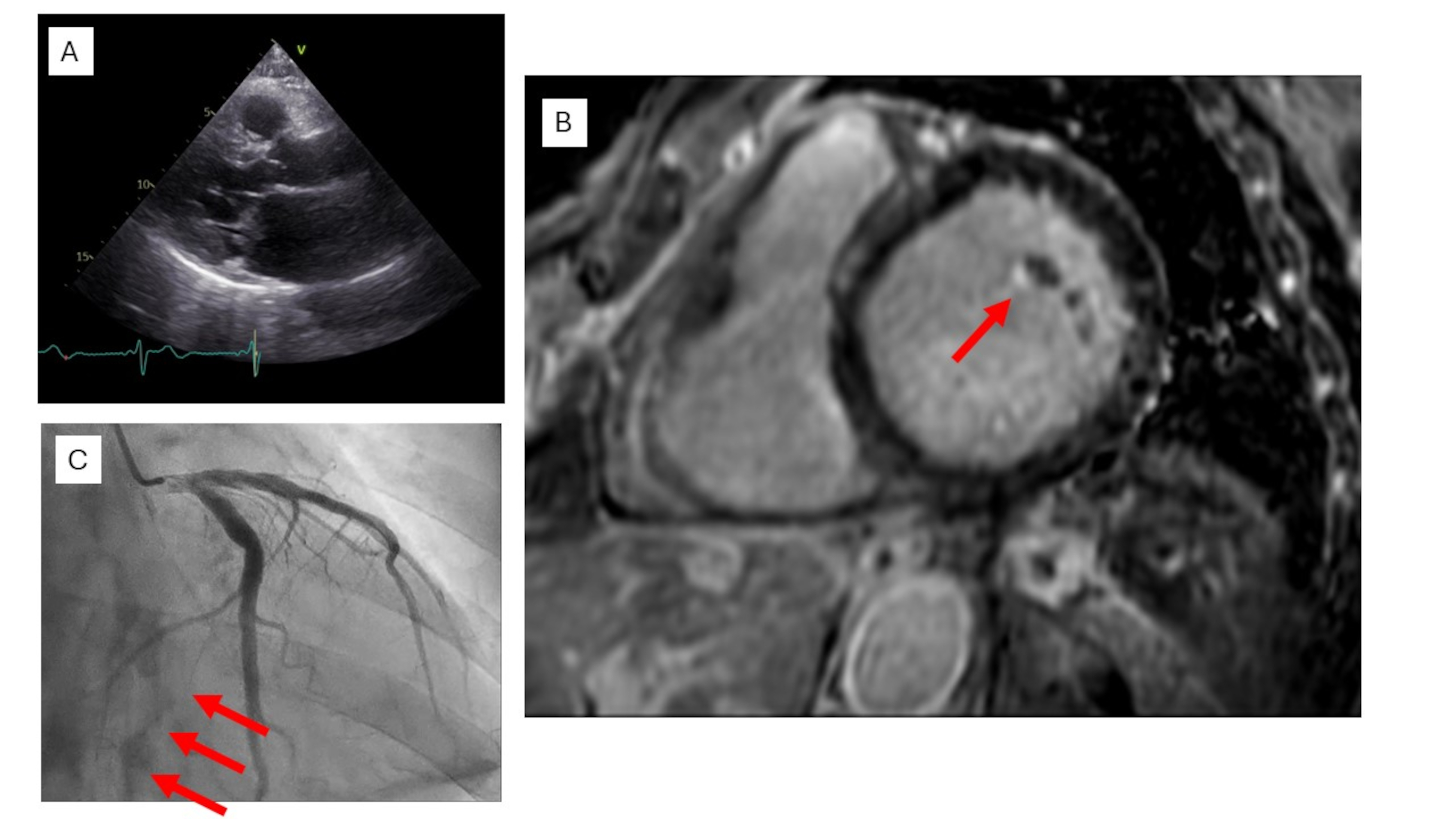

Supplement: ezaf461_Supplementary_Data [file ezaf461_Supplementary_Data.zip › Supplementary Figure 2 rev.JPG]

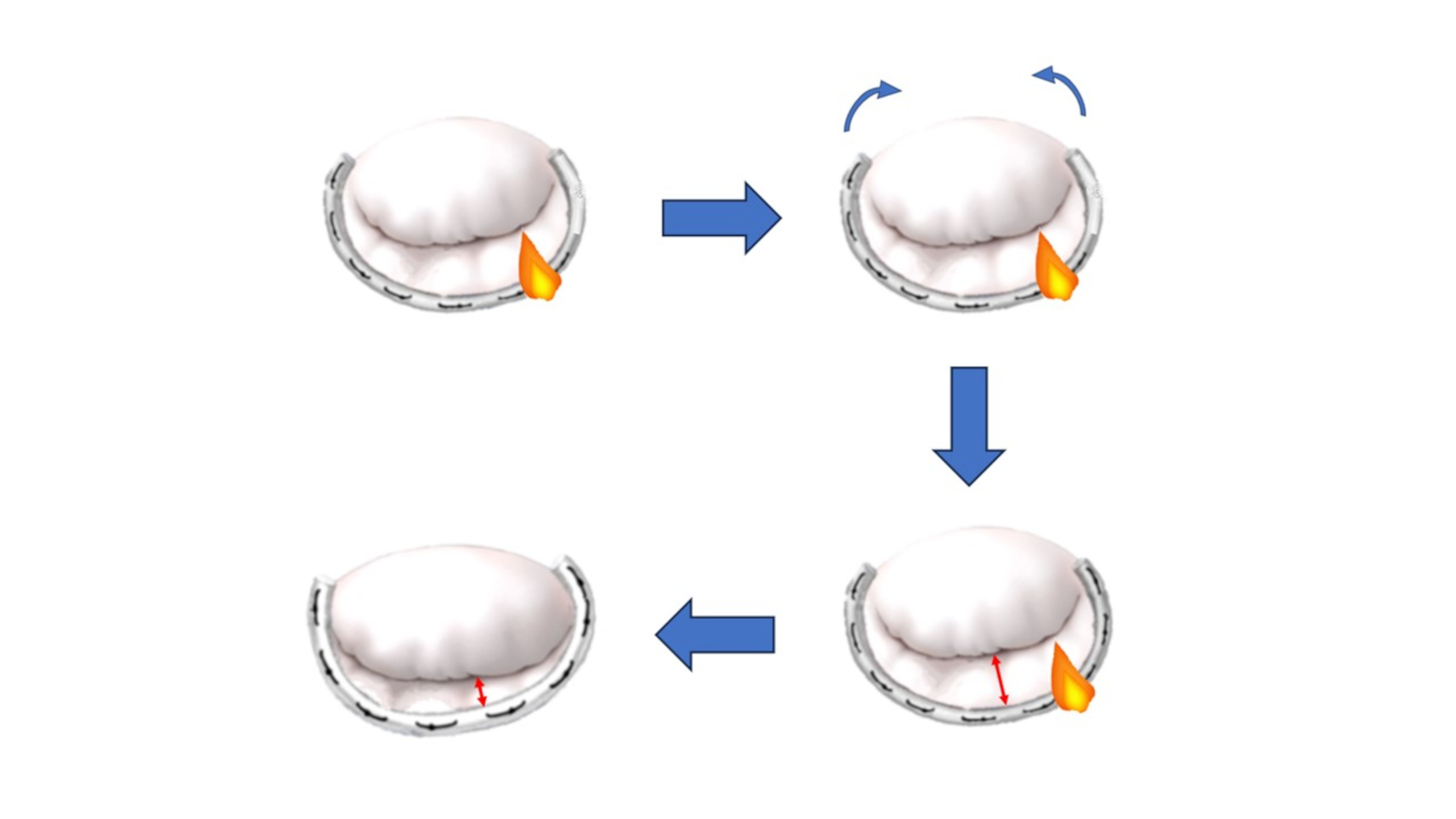

Supplement: ezaf461_Supplementary_Data [file ezaf461_Supplementary_Data.zip › Supplementary Figure 3 rev.JPG]
